# Macrophage RSAD2 Couples mtDNA Synthesis With a Self‐Amplifying Inflammatory Circuit to Orchestrate Tissue Repair

**DOI:** 10.1002/advs.77416

**Published:** 2026-08-29

**Authors:** Haomiao Yuan, Shukui Du, Chunfei Li, Xinjie Li, Shuyang Mu, Anran Qu, Hengxu Yan, Chongchong Xu, Jinnong Yang, Jingkai Sun, Yijun Wang, Jiayang Xiao, Fuyuan Zhang, Linlin Wang, Tiegang Li, Rui Zhao, Dawei Guan

**Affiliations:** ^1^ Department of Forensic Pathology China Medical University School of Forensic Medicine Shenyang China; ^2^ Liaoning Province Key Laboratory of Forensic Bio‐Evidence Science Shenyang China; ^3^ Department of Emergency Medicine Shengjing Hospital of China Medical University Shenyang China; ^4^ Department of Neurosurgery Shengjing Hospital of China Medical University Shenyang China; ^5^ Department of General Surgery Shengjing Hospital of China Medical University Shenyang China; ^6^ Institute of Forensic Science Fudan University Shanghai China; ^7^ Department of Pediatrics and Pediatric Epilepsy Center First Hospital of Peking University Beijing China; ^8^ China Key Laboratory of Environmental Stress and Chronic Disease Control & Prevention Ministry of Education China Medical University Shenyang China; ^9^ Department of Forensic Medicine Wenzhou Medical University School of Basic Medical Sciences Wenzhou China

**Keywords:** CMPK2, IFN response, inflammasome, macrophage, mitochondrial DNA, RSAD2, tissue repair

## Abstract

It is well established that mitochondrial DNA (mtDNA) synthesis governs macrophage function, yet its role in tissue repair and inflammation remains poorly understood. Using a mouse skin injury model combined with spatial transcriptomics and functional assays, we identify radical s‐adenosyl methionine domain containing 2 (RSAD2) as a critical regulator of mtDNA‐driven inflammation in macrophages. Mechanistically, RSAD2 directly binds the N‐terminal domain of cytidylate monophosphate kinase 2 (CMPK2), a rate‐limiting enzyme for mtDNA synthesis, and dually modulates its activity: it inhibits K63‐linked ubiquitination to stabilize CMPK2, and recruits casein kinase 2 alpha 2 (Csnk2a2) to promote CMPK2 phosphorylation, thereby enhancing mtDNA production. Newly synthesized mtDNA amplifies inflammation through two coordinated pathways: activation of a cGAS‐STING‐IRF3‐RSAD2 feedforward loop, and synergistic activation of the NLR family pyrin domain containing 3 (NLRP3) inflammasome. These pathways orchestrate inflammatory amplification in macrophages to modulate skin repair. Our work establishes macrophage RSAD2 as a central hub that coordinates two mtDNA‐dependent inflammatory axes, revealing a feedforward immuno‐metabolic circuit with broad implications for inflammation‐driven diseases.

## Graphical abstract

Macrophage RSAD2 dually regulates CMPK2—the rate‐limiting enzyme for mitochondrial DNA synthesis—by suppressing its ubiquitination and promoting its phosphorylation via Csnk2a2 recruitment. This amplifies mtDNA production, which simultaneously activates a cGAS‐STING‐IRF3‐RSAD2 feedforward loop and the NLRP3 inflammasome, driving a self‐sustaining inflammatory circuit that shapes tissue repair. Targeting RSAD2 disrupts this loop and accelerates wound healing.

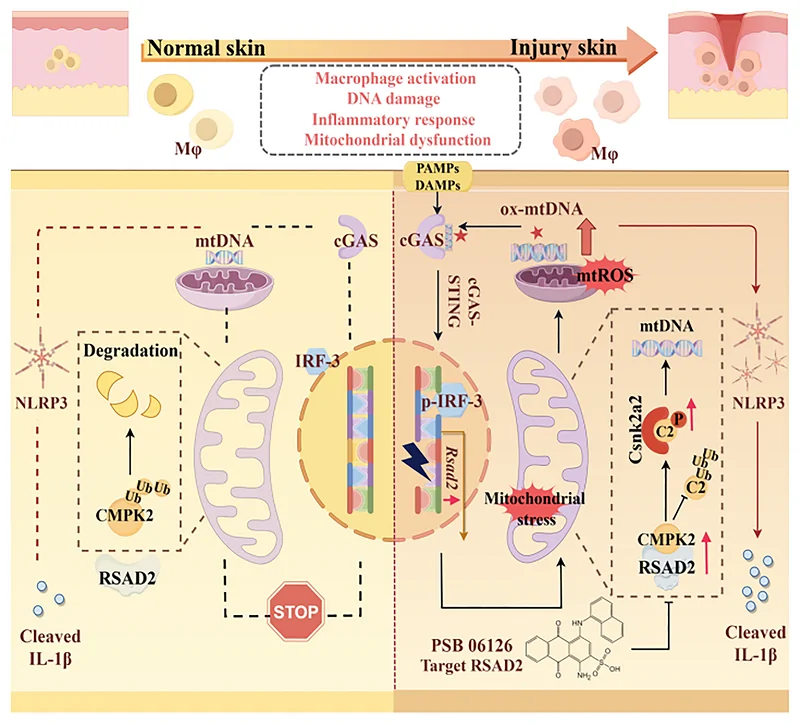

## 1 Introduction

Impaired wound healing and pathological scarring persist as global medical challenges, demanding novel therapeutic targets [1, 2]. The macrophage‐dependent transition from inflammation to repair following skin injury hinges critically on proinflammatory factors, particularly interleukin‑1β (IL‐1β) and the NLR family pyrin domain containing 3 (NLRP3) inflammasome [3, 4, 5]. NLRP3 activation requires a tightly regulated two‐step process: (nuclear factor κB) NF‐κB‐mediated priming and activation triggered by mitochondrial dysfunction, ionic imbalance, and ROS [6]. Critically, cytidylate monophosphate kinase 2 (CMPK2) ‐regulated mitochondrial DNA (mtDNA) release into the cytosol has been demonstrated as essential for NLRP3 inflammasome assembly, though upstream regulators of CMPK2 remain elusive [7, 8].

As the rate‐limiting enzyme providing deoxyribonucleotides for mtDNA synthesis, CMPK2 maintains mitochondrial nucleotide pool homeostasis by phosphorylating dCMP to dCDP [9]. Following tissue injury, elevated CMPK2 activity in macrophages promotes nascent mtDNA synthesis. Under mitochondrial ROS (mtROS) action, newly synthesized mtDNA is fragmented and converted into oxidized mtDNA (ox‐mtDNA), which translocates to the cytosol through the mitochondrial permeability transition pore (MPTP). This mislocalized ox‐mtDNA propagates inflammation via dual pathways [10, 11, 12]:(I) activating the mtDNA/NLRP3 axis to induce inflammasome assembly; and (II) triggering the mtDNA/cGAS‐STING‐IRF3 cascade to mediate interferon responses. Thus, mtDNA serves as a central hub linking mitochondrial dysfunction to coordinated inflammatory amplification, positioning CMPK2 as a promising therapeutic target for inflammation‐driven pathologies [13, 14, 15, 16].

Intriguingly, the interferon‐stimulated gene *radical s‐adenosyl methionine domain containing 2* (*Rsad2*) is renowned for its potent and broad‐spectrum antiviral functions [17]. As a typical interferon (IFN)‐inducible gene, *Rsad2* activation occurs through both IFN‐dependent and ‐independent pathways, with its expression regulated by the transcription factor interferon regulatory factor 3 (IRF3) [18, 19]. Recent studies reveal an undefined mechanistic link between RSAD2 and CMPK2 during inflammatory responses: (I) Coordinated transcriptional activation of *Rsad2* and *Cmpk2* upon IFN or pathogen‑associated molecular pattern stimulation [20, 21]. (II) highly co‐elevated levels of RSAD2 and CMPK2 among abundant proteins in M1 macrophages [22]. (III) Rapid upregulation of *Rsad2* mRNA during NLRP3 inflammasome activation [23, 24]. Collectively, these observations raise the possibility that RSAD2 may functionally cooperate with CMPK2 to modulate inflammation [25], yet the precise role of RSAD2 and the underlying molecular mechanisms remain unexplored.

Collectively, we demonstrate that following skin injury, upregulated RSAD2 in macrophages amplifies mtDNA‐mediated inflammation through direct interaction with CMPK2. Mechanistically, RSAD2 dually regulates CMPK2 by inhibiting its K63‐linked ubiquitination to enhance protein stability, and by recruiting casein kinase 2 alpha 2 (Csnk2a2) to promote CMPK2 phosphorylation, thereby boosting its catalytic activity and mtDNA synthesis. The resulting surge in mtDNA initiates a self‐amplifying inflammatory circuit: (I) activation of the cyclic GMP‑AMP synthase (cGAS)‐ stimulator of interferon genes (STING)‐IRF3 axis induces *Rsad2* transcription, establishing an mtDNA/cGAS‐STING‐IRF3‐RSAD2 feedforward loop; and (II) synergistic activation of the NLRP3 inflammasome amplifies inflammatory cascades. These two coordinated pathways orchestrate inflammatory responses in macrophages to modulate tissue repair. Our work repositions RSAD2 from a broad‐spectrum antiviral protein to a central immuno‐metabolic coordinator that bridges mitochondrial metabolism and nuclear transcription, revealing a feedforward circuit that integrates mtDNA‐driven cGAS and NLRP3 pathways with broad implications for inflammation‐driven diseases.

## 2 Results

### 2.1 RSAD2 is Highly Expressed in Macrophages During the Inflammatory Phase of Skin Injury

To investigate the cellular‐molecular networks in skin repair and identify novel targets, a comprehensive spatial transcriptomic atlas of skin injury and repair was constructed. Analysis revealed 11 cell clusters in wound regions (Figure 1A,B and Figure S1A,B), with spatiotemporal distributions consistent with repair progression [26] (Figure S1C). GO enrichment of differentially expressed genes (DEGs) further delineated repair dynamics. Cell–cell communication analysis demonstrated that macrophages (M1+M2) exhibited the highest outgoing signaling activity (Figure 1C,D and Figure S1D), suggesting their pivotal bridging role, while neutrophils showed dominant incoming signaling reception. Given the critical influence of inflammation on fibrotic/remodeling phases, inflammatory cell DEG heatmaps were generated (Figure 1G). *Rsad2* was identified as a key candidate gene, showing predominant expression in M1 macrophage SPOTs (Figure 1E,H,I) and ranking among the top 10 wound‐expressed genes at 12 h post‐injury (Figure 1F). Building upon omics data suggesting RSAD2's spatiotemporal expression specificity during skin repair, we validated its mRNA and protein distribution. Key findings include: (I) *Rsad2* mRNA peaked at 12h‐1d post‐injury, declined to baseline by day 5, and undetected at days 10–14 at the wound margin (Figure 1J). No significant changes were observed at peripheral wound zones. (II) RSAD2 protein dynamics mirrored mRNA expression patterns (Figure 1K,L). (III) RSAD2^+^ cells were absent in uninjured skin but abundant in wounds at 12h‐3d, disappearing by day 5 (Figure 1M). Additionally, KEGG enrichment in *Rsad2* ^+^ SPOTs revealed significant activation of NF‐κB, Toll‐like receptor, NOD‐like receptor, and cytosolic DNA‐sensing pathways (Figure S1E), indicating RSAD2's inflammatory role.

**FIGURE 1:**
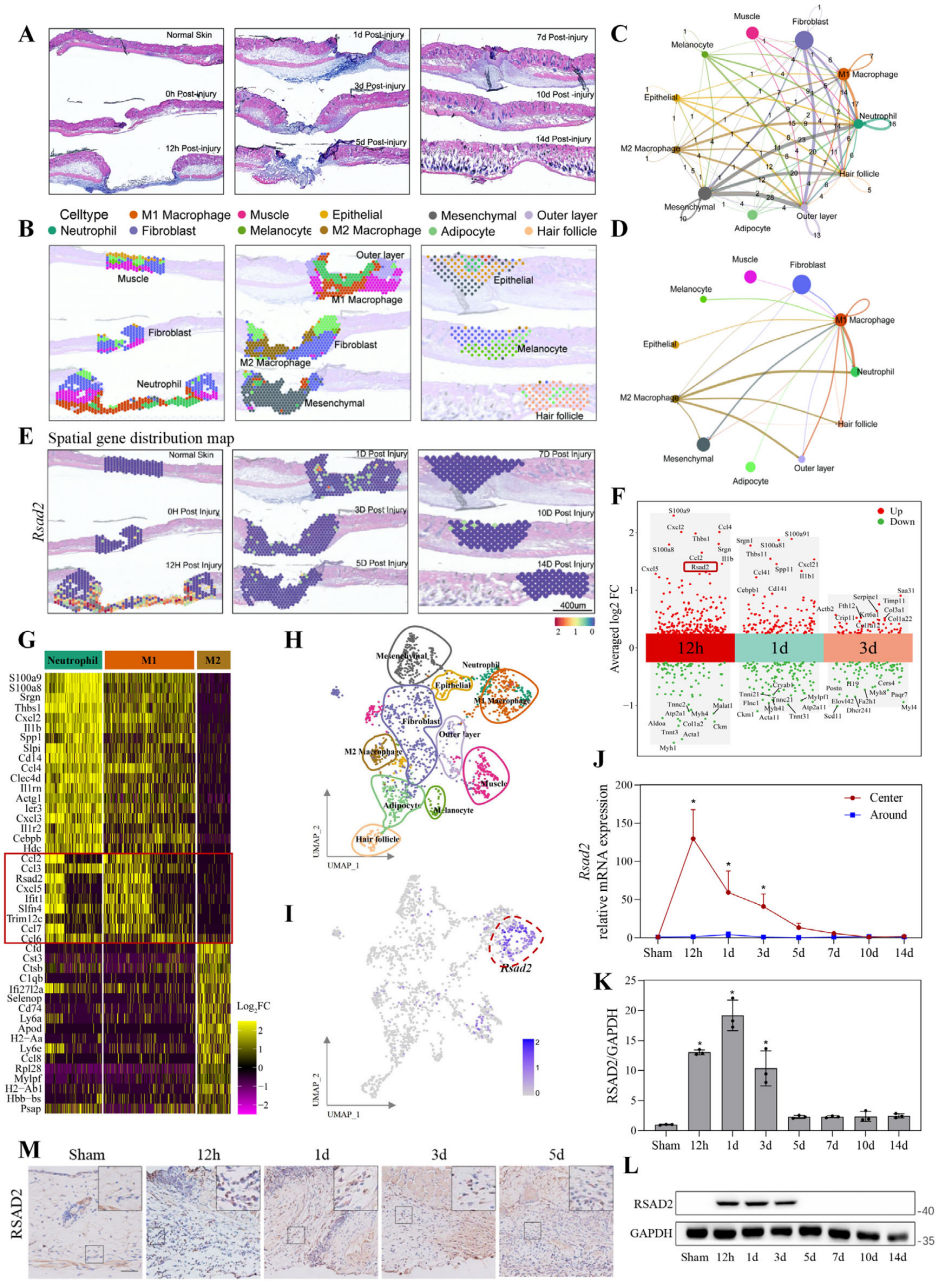
Spatial transcriptomic profiling of skin‑wound repair reveals spatiotemporal expression of RSAD2. (A) Representative H&E‑stained image of skin sections used for spatial transcriptomic analysis of wound repair. (B) Spatial distribution of major cell types across the wound area at indicated time points, shown as SPOT maps. (C) Ligand–receptor interaction network among all cell populations during skin wound and repair. (D) Ligand–receptor interaction network of macrophage subsets (M1 and M2) during skin wound and repair. (E) Spatial distribution map of *Rsad2* in skin tissues during skin wound and repair. (F) Volcano plots of differentially expressed genes at 12 h, 1 d, and 3 d after injury; red dots indicate upregulated genes, green dots indicate downregulated genes. (G) Heatmap showing differentially expressed genes within neutrophils and macrophages. (H, I) UMAP visualization of all cell clusters and *Rsad2* expression pattern across spatial transcriptomic data. (J) Line graph showing relative *Rsad2* mRNA expression in wound and peri‑wound regions over the healing process, normalized to the sham group, n = 5. (K, L) Immunoblot analysis of RSAD2 expression during skin wound and repair. Protein levels are normalized to the sham group, n = 3. (M) Representative immunohistochemical staining of RSAD2 in normal skin and wounded skin at 12 h, 1 d, and 3 d after injury, scale bar=200 µm. ^*^ *p* <0.05; NS, not significant.

To address spatial transcriptomics resolution constraints in single‐cell analysis, multicolor flow cytometry of skin mononuclear cells (SMNCs) was performed (Figure 2A). t‐SNE visualization revealed three dominant immune populations: neutrophils (light pink), macrophages (light blue), and T cells (light purple) (Figure 2B,C and Figure S1F). RSAD2^+^ cells (magenta) showed: (I) Primary overlap with macrophages at 12 h and 3d. (II) Partial overlap with T cells and neutrophils. MACS‑isolated F4/80^+^ macrophages from day 3 wounds further showed that RSAD2 expression was enriched in the CD86^+^ M1‑like subset, with lower expression in CD163^+^ or CD206^+^ M2‑like subsets (Figure S1G). Multiplex IHC (mIHC) corroborated these observations (Figure 2D,E): (I) Minimal CD11b^+^/Ly‐6G^+^ (neutrophils), CD11b^+^/F4/80^+^ (macrophages), or RSAD2^+^ cells in uninjured skin. (II) Higher CD11b^+^/F4/80^+^/RSAD2^+^ triple‐positive cells vs. CD11b^+^/Ly‐6G^+^ /RSAD2^+^ cells in injured skin. To further investigate the expression profile of RSAD2 in humans, we collected both injured skin (injury duration ≤ 3 days) and uninjured skin specimens. Western blot analysis revealed that RSAD2 exhibited minimal expression in normal human skin, while significantly expression following skin injury (Figure 2F,G). In vitro modeling using THP‐1 and HL‐60 cells showed that RSAD2 was significantly upregulated in differentiated THP‐1 macrophages upon LPS stimulation, but remained minimal in HL‐60 neutrophils regardless of stimulation (Figure 2H,I). Analysis of the human skin scRNA‑seq dataset GSE241132 revealed that RSAD2 expression was markedly increased in myeloid subsets upon injury, with predominant enrichment in macrophages (Figure 2J,K and Figure S1H). Pairwise analysis confirmed that the proportion of RSAD2^+^ cells was significantly higher in macrophages than in other myeloid lineages (Figure 2L). Collectively, these results demonstrate inflammation‐phase‐specific RSAD2 enrichment in macrophages during skin repair, conserved across mouse and human.

**FIGURE 2:**
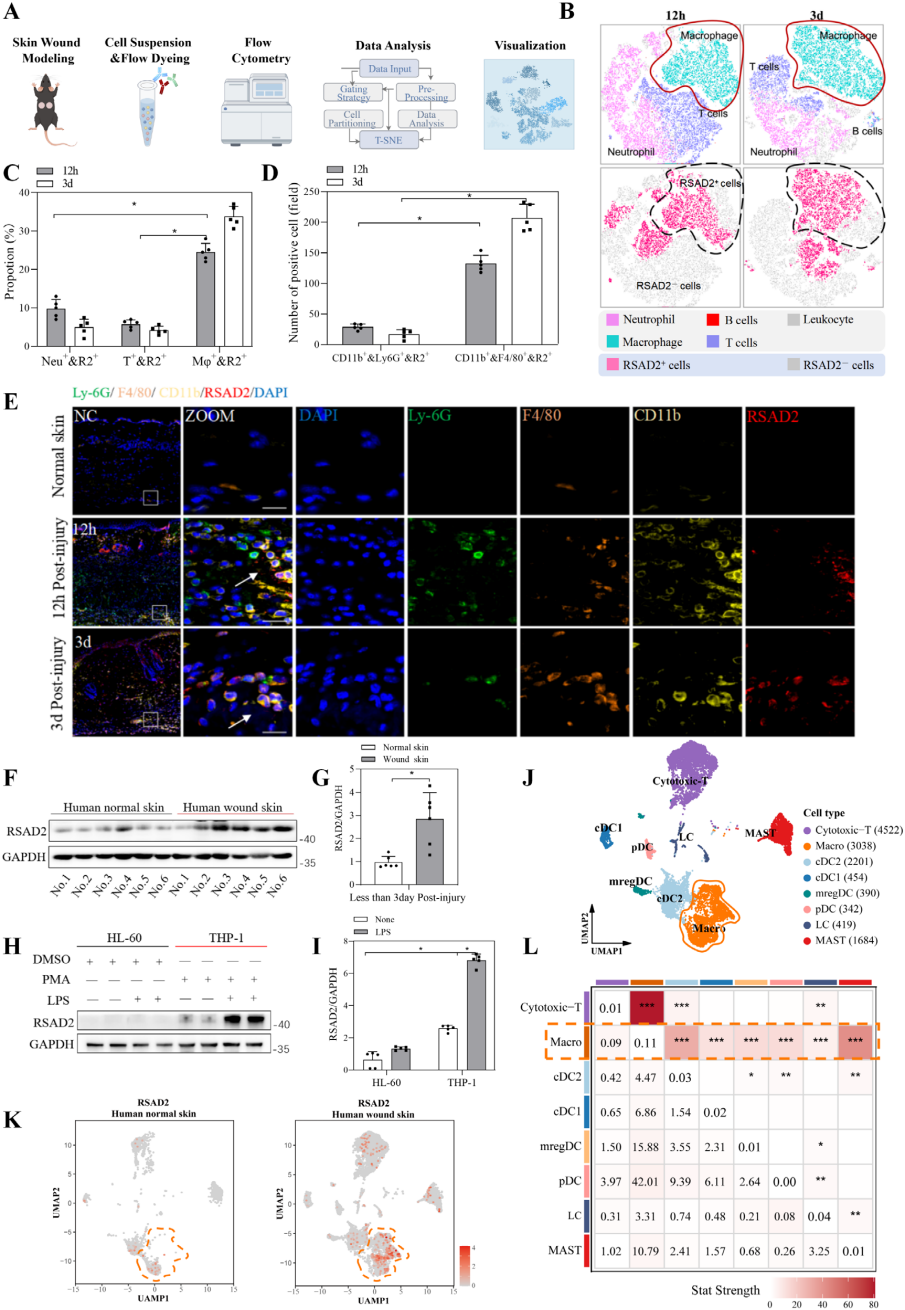
RSAD2 is highly expressed in macrophages During the inflammatory phase of skin‑wound repair. (A) Schematic workflow of multicolor flow cytometry analysis of skin mononuclear cells. (B, C) t‑SNE analysis and quantification of the proportion of RSAD2^+^ cells among neutrophils, T cells, and macrophages in wounded skin at 12 h and 3 d by flow cytometry. Each dot represents a single cell, colored by marker expression (n = 5). (D, E) Representative multiplex immunofluorescence images of RSAD2, Ly‑6G, CD11b, and F4/80 in normal and wounded skin at 12 h and 3 d, together with quantification of triple‑positive cells (CD11b^+^ F4/80^+^ RSAD2^+^ and CD11b^+^ Ly‑6G^+^ RSAD2^+^) in the wounds. Within‑group data were not compared; between‑group differences at single time points were analyzed using unpaired t tests (scale bar, 10 µm; n = 5). (F, G) Immunoblot analysis and quantification of RSAD2 protein expression in human normal skin and skin wounds (≤ 3 d after injury), analyzed by unpaired t test (n = 6). (H, I) Western blot analysis of RSAD2 expression in THP‐1 and HL‐60 cells after differentiation and LPS stimulation. Representative blots (H) and quantitative analysis (I) are shown. (J) UMAP dimensionality reduction of immune cell subsets (myeloid cells, lymphocytes, and mast cells) in human skin based on the GSE241132 dataset. Each dot represents a single cell, colored by cell type. (K) UMAP plots showing *Rsad2* expression in immune cell subsets from normal human skin (left) and wounded human skin (right) in the GSE241132 dataset. Color intensity indicates *Rsad2* expression levels. (L) Pairwise analysis of RSAD2 expression across immune cell subsets. Diagonal: percentage of RSAD2‐positive cells within each subset. Upper triangle: statistical significance of the comparison between RSAD2‐positive and RSAD2‐negative cell counts across subsets, determined by two‐tailed Fisher's exact test with Benjamini–Hochberg correction for multiple comparisons. Lower triangle: odds ratio for each corresponding comparison. Colors reflect the statistical significance level ^*^ *p* <0.05; NS, not significant.

### 2.2 Macrophage RSAD2 Expression Regulates Inflammatory Cell Recruitment and Fibrotic Repair After Skin Injury

To investigate RSAD2's role in skin wound and repair, myeloid‐specific *Rsad2* knockout (*Rsad2* ^−/−^) and AAV9‐CD68‐Rsad2^+^ overexpression mice models were established (Figure 3A and Figure S2A–I). Spatial transcriptomics revealed *Rsad2* ^+^ macrophage clusters in wound areas (Figure 3B) and PPI analysis of their top 20 DEGs suggested potential associations between RSAD2 and cytokines including IL‐1β, Cxcl2, Ccl4, and Ccl2 (Figure 3C), cytokine antibody array confirmed significantly reduced levels of GM‐CSF, IL‐1β, IL‐6, Cxcl1, Cxcl5, and Ccl3 in *Rsad2* ^−/−^ mice wounds vs. *Rsad2* ^fl/fl^ (Figure 3D). RT‐qPCR and ELISA further validated that *Rsad2* deficiency reduced IL‐1β and IL‐6 expression, while RSAD2 overexpression increased proinflammatory cytokine production (Figure 3E and Figure S2J–M).

**FIGURE 3:**
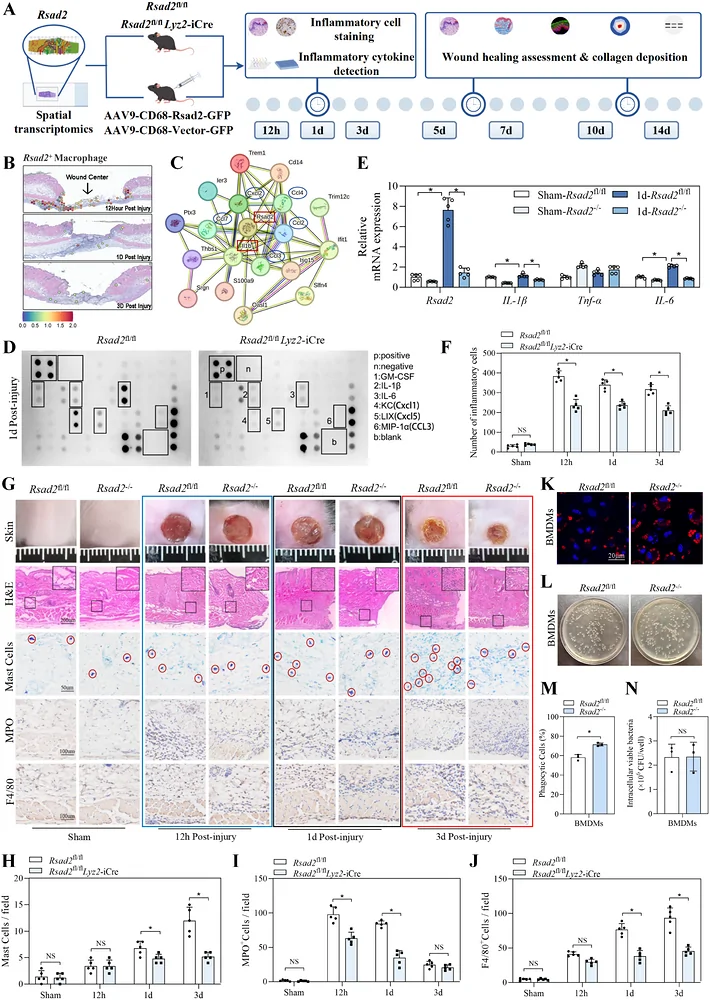
RSAD2 promotes inflammatory cell infiltration in wounded skin. (A) Schematic diagram of the experimental design for investigating RSAD2 function during skin wound repair. (B) Spatial distribution of *Rsad2* ^+^ Macrophage SPOTs in the spatial transcriptomic map of skin wound repair. (C) Protein–protein interaction network of the top 20 upregulated genes in *Rsad2* ^+^ Macrophage SPOTs. (D) Cytokine/chemokine antibody microarray analysis of skin tissue from *Rsad2* ^fl/fl^ and *Rsad2* ^−/−^ mice at 1 d after injury. (E) Quantification of mRNA levels of three proinflammatory cytokines in the wound tissue of *Rsad2* ^fl/fl^ and *Rsad2* ^−/−^ mice at 1 d after injury. (F, G, H, I, J) Representative H&E, toluidine blue (mast cell staining), MPO, and F4/80 IHC images showing inflammatory cell infiltration in normal skin and wound areas at 12 h, 1 d, and 3 d after injury in *Rsad2* ^fl/fl^ and *Rsad2* ^−/−^ mice, along with quantification of total inflammatory cells, neutrophils, macrophages, and mast cells in the indicated groups (n = 5). (K) Representative confocal images of the phagocytosis assay using pHrodo Red Zymosan A BioParticles in *Rsad2*fl/fl and *Rsad2* ^−/−^ BMDMs. Scale bar = 200 µm. (L) Representative images of LB agar plates from the gentamicin protection assay showing intracellular *E. coli* colonies recovered from *Rsad2* ^fl/fl^ and *Rsad2* ^−/−^ BMDMs. (M) Quantification of the percentage of phagocytic macrophages in *Rsad2* ^fl/fl^ and *Rsad2* ^−/−^ BMDMs (n = 3). (N) Quantification of intracellular viable bacteria (CFU/well) in *Rsad2* ^fl/fl^ and *Rsad2* ^−/−^ BMDMs after the gentamicin protection assay (n = 3). Within‐group comparisons were analyzed using unpaired t tests. ^*^ *p* <0.05; NS, not significant.

Given cytokines' critical role in immune cell recruitment and activation, histological analysis of inflammatory infiltration revealed that *Rsad2* ^−/−^ mice exhibited reduced total inflammatory cells, neutrophils, macrophages, and mast cells in wounds at early time points, whereas AAV‐Rsad2^+^ mice showed enhanced macrophage infiltration at 3d post‐injury (Figure 3F–J and Figure S2N–R). Notably, this reduced inflammatory tone did not compromise macrophage host defense; RSAD2 deficiency enhanced phagocytic uptake without affecting bactericidal capacity (Figure 3K–N). These findings demonstrate that macrophage RSAD2 expression modulates cytokine‐driven inflammatory cell recruitment during skin repair without impairing innate immune defense functions.

Since skin wound healing requires coordinated transition from inflammation to remodeling, we next examined the impact of RSAD2‐mediated inflammation on the subsequent proliferative and remodeling phases. Morphometric analysis demonstrated significantly accelerated wound closure in *Rsad2* ^−/−^ mice but delayed healing in AAV‐Rsad2^+^ mice (Figure S3A,B). To assess healing quality during the proliferative phase, we evaluated re‐epithelialization, angiogenesis, and granulation tissue formation at day 7 post‐injury. H&E staining and CD31 immunofluorescence revealed that *Rsad2* ^−/−^ mice exhibited longer epithelial tongues, increased CD31^+^ vessel density, and thicker granulation tissue compared to *Rsad2* ^fl/fl^ controls, whereas AAV‐Rsad2^+^ mice showed opposite trends (Figure S3C–F). At day 10 post‐injury, Masson's trichrome and Collagen I immunofluorescence staining revealed more organized collagen deposition and reduced fibrosis in *Rsad2* ^−/−^ wounds (Figure S3G). Western blot analysis confirmed decreased expression of collagen I, fibronectin, and vimentin in *Rsad2* ^−/−^ wounds, indicating reduced ECM deposition (Figure S3H). H&E staining showed nearly normalized epidermal and dermal thickness in *Rsad2* ^−/−^ mice, contrasting with thickened scarring in *Rsad2* ^fl/fl^ and AAV‐Rsad2^+^ mice (Figure S3I–K). Collectively, these results establish that RSAD2‐mediated inflammation critically influences both early immune cell recruitment, proliferative repair, and subsequent fibrotic remodeling during skin wound healing.

### 2.3 Macrophage RSAD2 Modulates Inflammatory Response via NLRP3 Inflammasome Activation During Skin Repair

To elucidate pathways mediating RSAD2‐dependent cytokine secretion and immune cell recruitment, KEGG and GSEA analyses were performed on the top 20 DEGs from *Rsad2* ^+^ macrophage SPOTs. Key findings revealed that the top 20 DEGs significantly enrich in cytokine‐receptor interaction, Toll‐like receptor, and NOD‐like receptor (Figure S4A), among which NOD‐like receptor signaling distinctly upregulated in *Rsad2* ^+^ macrophage SPOTs (Figure 4A). Given RSAD2's profound impact on IL‐1β secretion (established by aforementioned results) and IL‐1β’s primary dependence on NLRP3 inflammasome activation, we investigated RSAD2‐NLRP3 interplay from multiple dimensions. (I) Spatiotemporal correlation: *Rsad2* and *Nlrp3* mRNA co‐localized in wound regions at 12 h, 1d, and 3d post‐injury through spatial distribution analysis (Figure 4B). Similarly, immunofluorescence confirmed RSAD2‐NLRP3 obvious co‐localization within wound macrophages (Figure 4C,D). (II) Protein dynamics: Western blotting revealed parallel transient upregulation of RSAD2 and NLRP3. Correlation analysis demonstrated that strong positive correlation between protein levels (Figure 4E). These results demonstrate spatiotemporal coordination between RSAD2 and NLRP3, indicating potential regulatory interactions.

**FIGURE 4:**
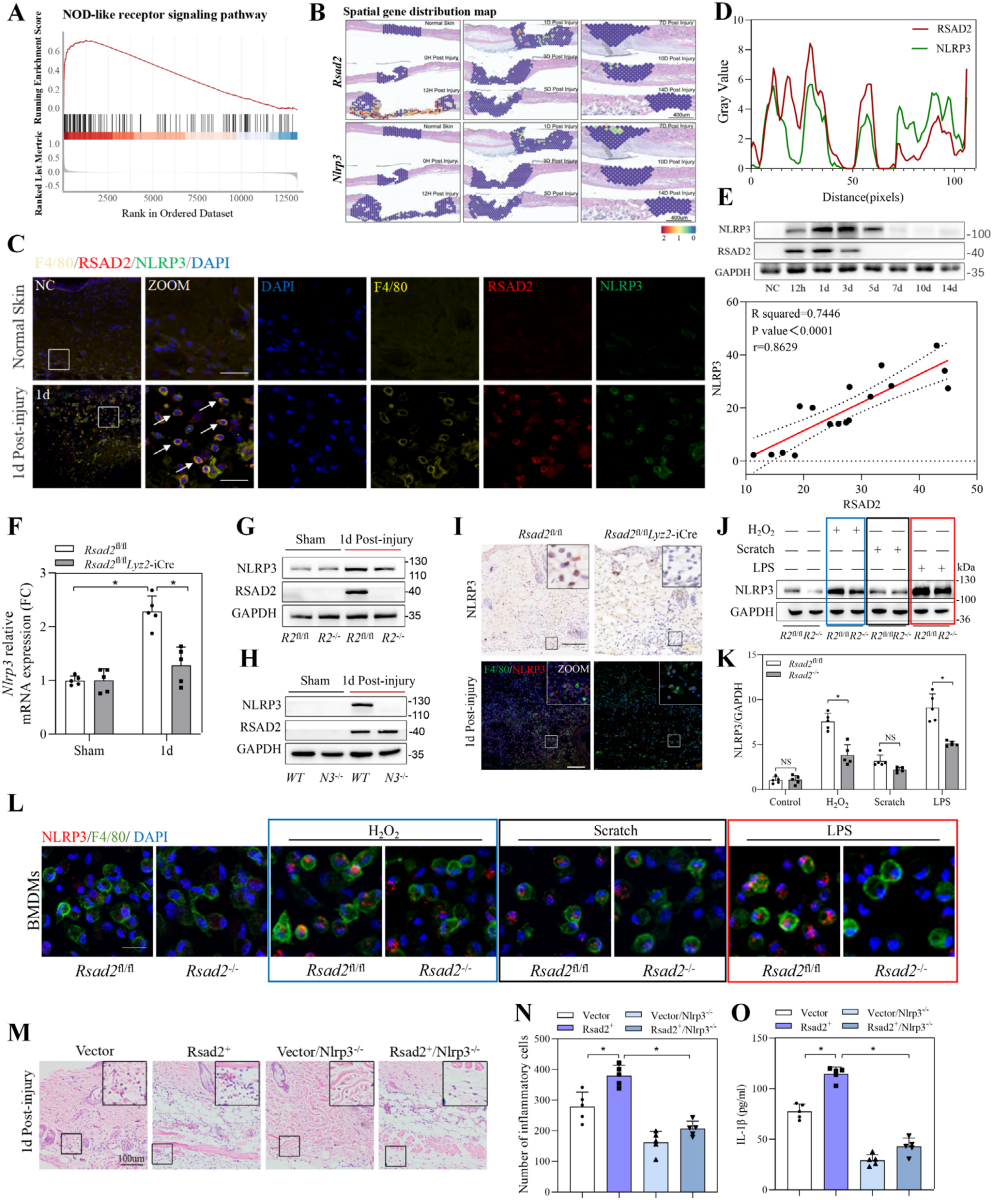
Macrophage RSAD2 modulates skin wound inflammation by regulating the NLRP3 inflammasome. (A) Gene‑set enrichment analysis (GSEA) of the NOD‑like receptor signaling pathway. (B) Spatial SPOT distribution of *Rsad2* and *Nlrp3* mRNA in the skin wound repair transcriptomic map. (C, D) Representative immunofluorescence images of F4/80, RSAD2, and NLRP3 in the wound area of normal and 1‑day‑injured skin, together with colocalization analysis of RSAD2 and NLRP3 within macrophages (scale bar, 50 µm). (E) Immunoblot analysis of RSAD2 and NLRP3 protein expression during skin wound repair and correlation between their levels. (F) Quantification of *Nlrp3* mRNA expression in skin tissues of *Rsad2* ^fl/fl^ and *Rsad2* ^−/−^ mice under normal conditions and at 1 day post‐injury (n = 5). (G) Representative immunoblots showing RSAD2 and NLRP3 protein expression in normal and 1‑day‑injured skin of *Rsad2* ^fl/fl^ and *Rsad2* ^−/−^ mice. (H) Representative immunoblots showing RSAD2 and NLRP3 protein expression in normal and 1‑day‑injured skin of *WT* and *Nlrp3* ^−/−^ mice. (I) Representative immunohistochemical staining of NLRP3 and immunofluorescence co‑staining of F4/80 and NLRP3 in skin wounds from *Rsad2* ^fl/fl^ and *Rsad2* ^−/−^ mice at 1 day after injury (scale bar, 100 µm). (J, K) NLRP3 protein expression levels in BMDMs from *Rsad2* ^fl/fl^ and *Rsad2* ^−/−^ mice after LPS, H_2_O_2_, or scratch stimulation (n = 5). (L) Immunocytochemical detection of NLRP3 in BMDMs from *Rsad2* ^fl/fl^ and *Rsad2* ^−/−^ mice after LPS, H_2_O_2_, or scratch stimulation (scale bar, 20 µm). (M, N, O) Representative H&E staining images and quantification of inflammatory cell infiltration and IL‑1β secretion in wound tissues from mice treated with AAV9‐CD68‐Vector, AAV9‐CD68‐Rsad2^+^, AAV9‐CD68‐ Vector/*Nlrp3* ^−/−^, and AAV9‑CD68‑Rsad2^+^/*Nlrp3* ^−/−^ at 1 day after injury (n = 5; scale bar, 100 µm). Within‐group comparisons were analyzed using unpaired t tests; between‑group differences were determined by two‐way ANOVA followed by Tukey's post‑hoc test. ^*^ *p* <0.05; NS, not significant.

To delineate the RSAD2‐NLRP3 regulatory mode, we employed *Rsad2* ^fl/fl^, *Rsad2* ^−/−^, *Nlrp3* ^−/−^ mice (Figure S4B), *WT* and *Nlrp3* ^−/−^ mice receiving AAV9‐CD68‐vector or AAV9‐CD68‐Rsad2^+^. Western blot analysis revealed reduced NLRP3 expression in *Rsad2* ^−/−^ mice vs. *Rsad2* ^fl/fl^ (Figure 4G), whereas RSAD2 expression remained unchanged in *Nlrp3* ^−/−^ mice vs. *WT* (Figure 4H). This positions RSAD2 upstream of NLRP3 signaling, further supported by RT‐qPCR (Figure 4F). IHC and immunofluorescence co‐localization confirmed a decrease in the number of NLRP3^+^ macrophages in wounds (Figure 4I). In vitro modeling, BMDMs from *Rsad2* ^fl/fl^ and *Rsad2* ^−/−^ mice showed concordant results by immunocytochemistry (ICC) and Western blot (Figure 4J–L). Reverse validation was further conducted. H&E staining showed that the prominent monocyte infiltration and inflammatory response in AAV‐Rsad2^+^ mice were largely abolished by *Nlrp3* deficiency, with infiltration levels in AAV‐Rsad2^+^/*Nlrp3* ^−/−^ mice comparable to those in AAV‐Vector/*Nlrp3* ^−/−^ and WT controls (Figure 4M,N). ELISA and Western blot analyses further confirmed these observations (Figure 4O, Figure S4C). Collectively, these results demonstrate that NLRP3 deficiency attenuates the pro‐inflammatory effects of RSAD2 overexpression, indicating that RSAD2 promotes inflammatory responses through the NLRP3 inflammasome.

### 2.4 Macrophage RSAD2 Regulates NLRP3 Inflammasome Activation via the CMPK2/mtDNA Pathway

Although our data demonstrate RSAD2‐dependent NLRP3 modulation, the molecular mechanism remained undefined. NLRP3 inflammasome activation requires two‐stage regulation, which includes priming signal and an activation signal [6, 27]. Using macrophages from *Rsad2* ^fl/fl^ and *Rsad2* ^−/−^ mice, we assessed RSAD2 impact on both stages. For the priming signal, Western blot revealed reduced NF‐κB and phospho‐NF‐κB in stimulated *Rsad2* ^−/−^ macrophages vs. *Rsad2* ^fl/fl^ (Figure S4D,E), consistent with prior findings [28, 29]. However, reduced priming alone does not necessarily impair inflammasome activation: under LPS‑alone conditions, although NLRP3 expression was reduced in *Rsad2* ^−/−^ BMDMs, neither cleaved caspase‑1 nor cleaved IL‑1β/IL‑18 was detected in cell lysates or culture supernatants, regardless of RSAD2 deficiency (Figure S4F–H). Only upon LPS+ATP stimulation did robust caspase‑1 cleavage and IL‑1β/IL‑18 secretion become apparent, with RSAD2 deficiency significantly attenuating these responses. Consistently, ASC speck formation was also markedly reduced in *Rsad2* ^−/−^ BMDMs upon LPS+ATP stimulation (Figure S4I,J). These results confirm that an intact activation signal is required for inflammasome function and that RSAD2 specifically regulates this step. To dissect this further, for activation signals, lysosomal function evaluated by DQ‐BSA assays showed unaltered lysosomal permeability and degradation capacity in *Rsad2* ^−/−^cells vs. *Rsad2* ^fl/fl^ (Figure 5A and Figure S4K–M). Calcium flux was indicated persistent Ca^2^^+^ elevation post‐stimulation regardless of RSAD2 status (Figure 5B, and Figure S4N). Nevertheless, we found RSAD2 significantly impacts mitochondrial homeostasis. In stimulated *Rsad2* ^−/−^ macrophages vs. *Rsad2* ^fl/fl^, total ROS and mtROS significantly were reduced (Figure 5C,D and Figure S4O), and loss of mitochondrial membrane potential (Δψ) attenuated (Figure 5E and Figure S4P). Similarly, transmission electron microscopy revealed a decrease in the number of condensed, and structurally indistinct mitochondria in injured macrophage following RSAD2 depletion (Figure 5F). As introduced, CMPK2 serves as the rate‐limiting enzyme for deoxyribonucleotide provision during mtDNA synthesis, an essential step for NLRP3 activation. Upon stimulation, macrophage mitochondrial destabilization facilitates mtROS‐dependent conversion of mtDNA to oxidized mtDNA (ox‐mtDNA). We next examined CMPK2/mtDNA regulation by RSAD2 in stimulated macrophages. mtDNA quantification demonstrated that RSAD2 deficiency attenuated mtDNA synthesis (Figure 5G,H). The comet assay showed that RSAD2 deficiency reduced mtDNA fragmentation, as quantified by tail moment (Figure 5I). Additionally, measurement of 8‐OHdG via ELISA following subcellular fractionation indicated decreased cytosolic ox‐mtDNA in *Rsad2* ^−/−^ macrophages (Figure 5J). However, MPTP assessment indicated unaltered MPTP‐mediated mitochondrial permeabilization regardless of RSAD2 expression (Figure 5K and Figure S4V).

**FIGURE 5:**
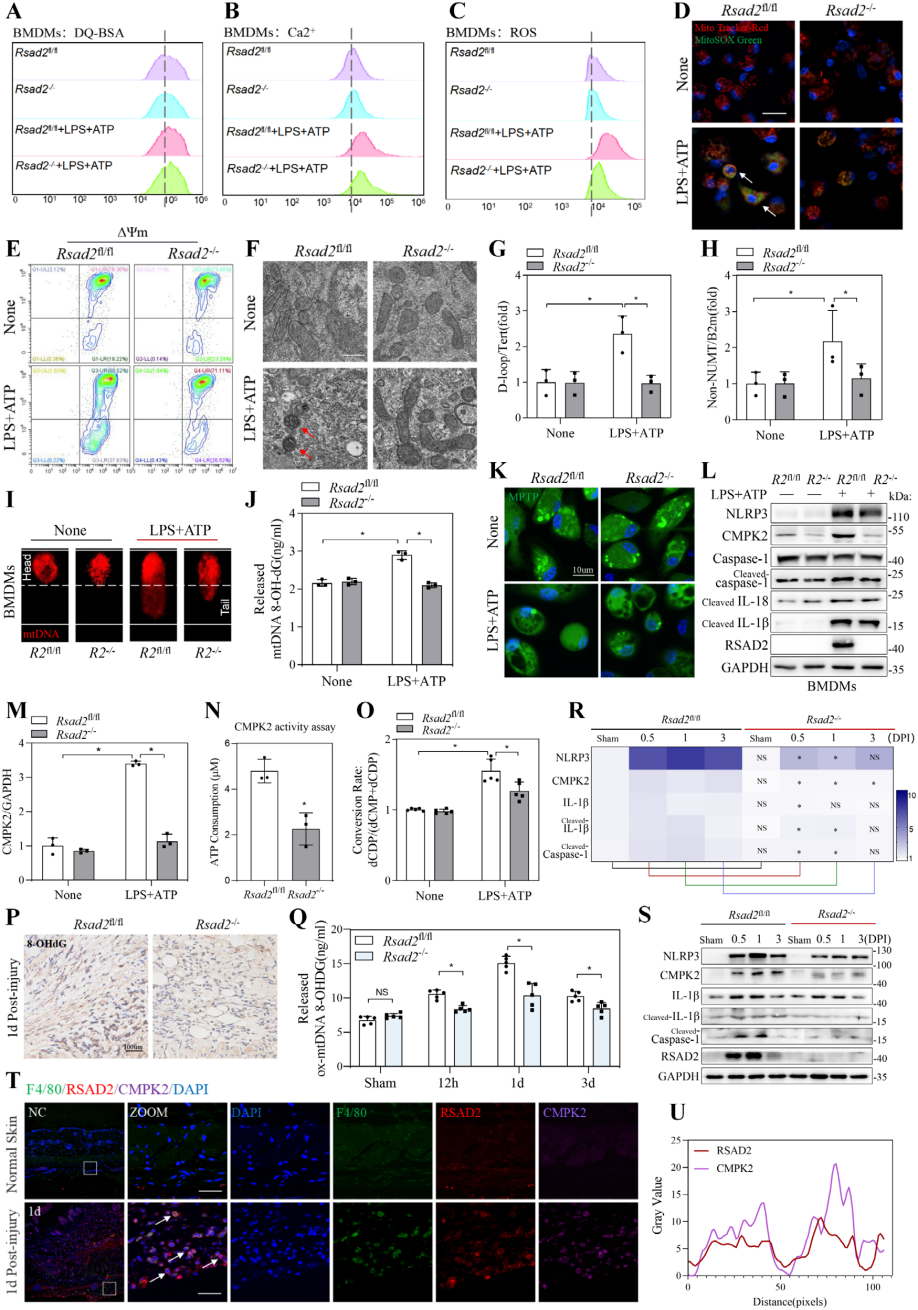
RSAD2 regulates NLRP3 inflammasome activation in macrophages through the CMPK2/mtDNA pathway. (A) Flow‐cytometric analysis of DQ‐BSA fluorescence in *Rsad2* ^fl/fl^ and *Rsad2* ^−/−^ BMDMs before and after LPS+ATP stimulation (n = 3). (B) Flow‐cytometric analysis of intracellular Ca^2+^ levels (n = 3). (C) Flow‑cytometric analysis of ROS production (n = 3). (D) Representative confocal images of MitoSOX and MitoTracker‑Red staining in *Rsad2* ^fl/fl^ and *Rsad2* ^−/−^ BMDMs before and after LPS+ATP stimulation (scale bar, 10 µm). (E) Flow‑cytometric assessment of mitochondrial membrane potential using JC‑1 staining from *Rsad2* ^fl/fl^ and *Rsad2* ^−/−^ BMDMs before and after LPS+ATP stimulation. (F) Representative transmission electron microscopy images of BMDMs from *Rsad2* ^fl/fl^ and *Rsad2* ^−/−^ BMDMs before and after LPS+ATP stimulation. (G, H) qPCR analysis showing the copy number of D‐loop (normalized to Tert) and non‐NUMT regions (normalized to B2m) in *Rsad2* ^fl/fl^ and *Rsad2* ^−/−^ BMDMs before and after LPS+ATP stimulation. (I) Representative confocal images of mtDNA comet assay in *Rsad2* ^fl/fl^ and *Rsad2* ^−/−^ BMDMs before and after LPS+ATP stimulation. (J) Measurement of cytosolic 8‑OHdG content by ELISA in *Rsad2* ^fl/fl^ and *Rsad2* ^−/−^ BMDMs before and after LPS+ATP stimulation. (K) Representative confocal images showing MPTP staining before and after stimulation (scale bar, 10 µm). (L, M) Immunoblot and quantitative analysis of CMPK2, NLRP3, and RSAD2 protein levels in *Rsad2* ^fl/fl^ and *Rsad2* ^−/−^ BMDMs (n = 3). (N) Quantification of ATP consumption by in vitro kinase assays for CMPK2 from *Rsad2* ^fl/fl^ and *Rsad2* ^−/−^ BMDMs before and after stimulation (n = 5). (O) Measurement of dCMP and dCDP levels by ELISA in BMDMs before and after LPS + ATP stimulation. Data were normalized to the untreated *Rsad2* ^fl/fl^ group (set to 1), and the catalytic conversion rate of dCDP was calculated as dCDP / (dCMP + dCDP). (P) Representative immunohistochemical staining of 8‐OHdG in wound areas of *Rsad2* ^fl/fl^ and *Rsad2* ^−/−^ mice at 1 day after injury (scale bar, 100 µm). (Q) Assessment of cytosolic 8‑OHdG levels in F4/80^+^ macrophages from *Rsad2* ^fl/fl^ and *Rsad2* ^−/−^ mice at 12 h, 1 d, and 3 d after injury, measured by ELISA (n = 5). (R, S) Immunoblot and heatmap quantification of CMPK2, RSAD2, and NLRP3 protein expression in normal skin and at 12 h, 1 d, and 3 d after injury in *Rsad2* ^fl/fl^ and *Rsad2* ^−/−^ mice. Protein expression was normalized to the *Rsad2* ^fl/fl^ sham group (n = 3). (T, U) Representative immunofluorescence images of F4/80, RSAD2, and CMPK2 in normal skin and skin wounds at 1 day after injury, together with colocalization analysis in the wound area (scale bar, 20 µm). Within‐group comparisons were analyzed using unpaired t tests; between‑group differences were determined by two‐way ANOVA followed by Tukey's post‑hoc test. ^*^ *p* <0.05; NS, not significant.

Owing to the central role of CMPK2, we evaluated its protein expression and catalytic activity. Western blot analysis revealed that RSAD2 deficiency significantly reduced CMPK2 expression (Figure 5L,M). Kinase assays in vitro showed that RSAD2‐ablated macrophages exhibited reduced ATP consumption during CMPK2‐mediated reactions, indicating diminished catalytic activity (Figure 5N). ELISA further demonstrated that RSAD2 ablation reduced the catalytic efficiency of CMPK2 toward its substrate dCMP compared to *Rsad2* ^fl/fl^ (Figure 5O and Figure S4Q,R).

For validation of the RSAD2‐CMPK2/mtDNA/NLRP3 axis in vivo, we performed immunohistochemistry and immunofluorescence analyses of skin wounds. RSAD2 deficiency reduced the number of 8‐OHdG^+^ cells in wounds (Figure 5P) and decreased cytosolic 8‐OHdG content in skin F4/80^+^ cells (Figure 5Q). Western blot analysis of injured skin showed significantly reduced levels of CMPK2, NLRP3, IL‐1β, cleaved IL‐1β, and cleaved caspase‐1 in *Rsad2* ^−/−^ vs. *Rsad2* ^fl/fl^ mice, indicative of suppressed NLRP3 activation (Figure 5R,S). The mtDNA levels in F4/80^+^ cells of injured skin was indicated a decreased mtDNA copy number in *Rsad2* ^−/−^ vs. *Rsad2* ^fl/fl^ mice (Figure S4S,T). Immunofluorescence co‐localization analysis revealed enhanced RSAD2 and CMPK2 expression with intensified co‐localization in macrophages (Figure 5T,U). Correlation analysis further demonstrated a significant positive correlation between *Rsad2* and *Cmpk*2 mRNA levels by spatial distribution map (Figure S4W), and protein levels by western blot (r = 0.9380, R^2^ = 0.8798, *p* <0.0001; Figure S4X,Y). These findings establish RSAD2 as a key regulator of macrophage‐driven inflammatory responses through the CMPK2/mtDNA/NLRP3 cascade, and a strong correlation of RSAD2‐CMPK2.

### 2.5 RSAD2 Translocates to Mitochondria and Interacts With CMPK2^1∼257aa^

Our data establish that CMPK2 expression is regulated by RSAD2, but the precise mechanism remains unknown. RT‐qPCR revealed no significant reduction in *Cmpk2* mRNA upon RSAD2 ablation (Figure 6A), excluding transcriptional regulation. We therefore examined post‐translational modifications and potential direct interaction between RSAD2 and CMPK2.

**FIGURE 6:**
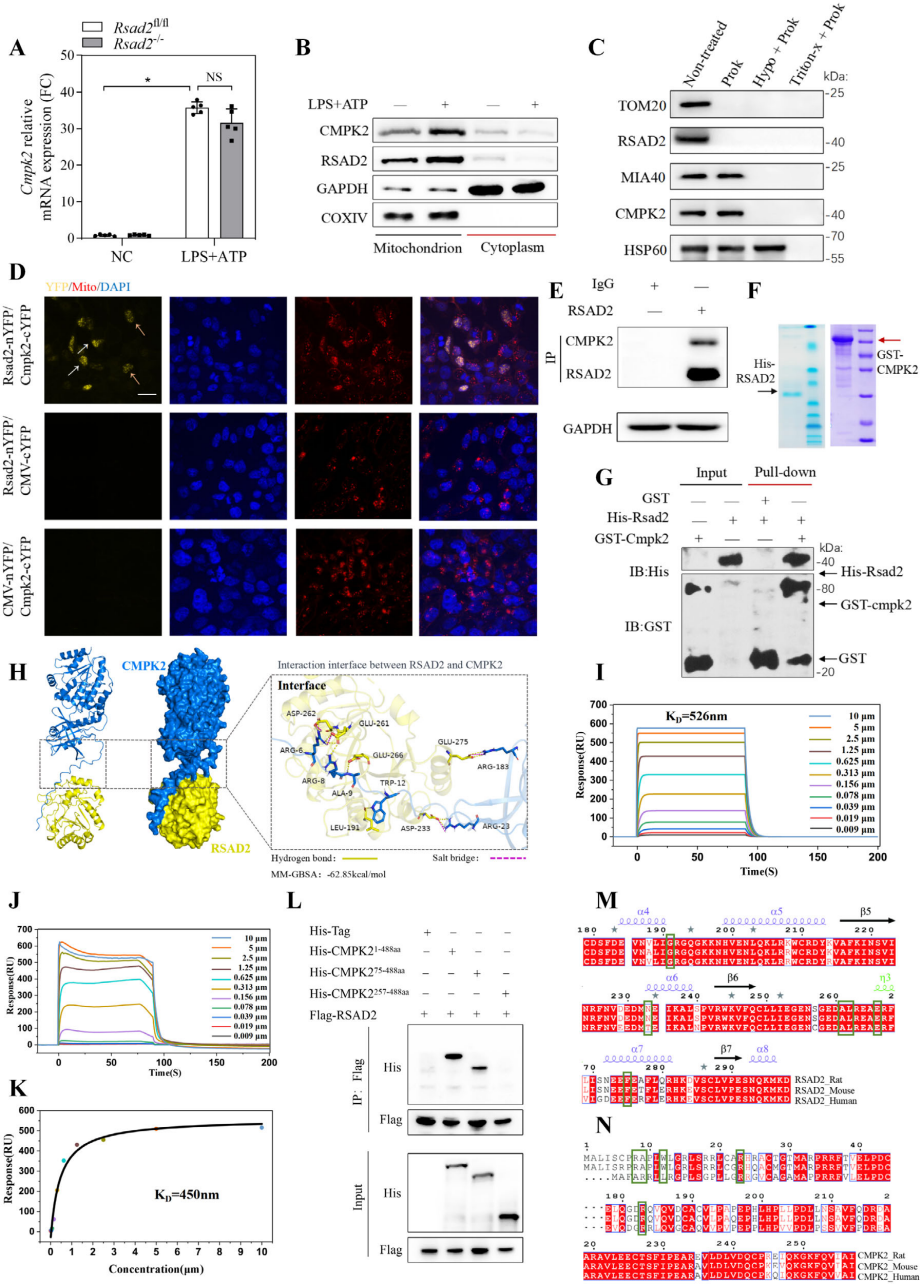
RSAD2 translocates to mitochondria and interacts with CMPK2^1∼257aa^ (A) Quantification of *Cmpk2* mRNA levels in *Rsad2* ^fl/fl^ and *Rsad2* ^−/−^ macrophages before and after LPS+ATP stimulation. Within‐group comparisons were analyzed using unpaired t tests; between‑group differences were determined by two‐way ANOVA followed by Tukey's post‐hoc test (n = 5). (B) Representative immunoblots of RSAD2 and CMPK2 in mitochondrial and cytosolic fractions of BMDMs before and after LPS+ATP stimulation. (C) Immunoblot analysis of RSAD2, CMPK2, TOM20, MIA40, and HSP60 in purified mitochondrial fractions treated with ProK alone, hypotonic buffer + ProK, or Triton X‐100 + ProK. (D) Immunocytochemical detection of RSAD2–CMPK2 interaction in transfected HEK‐293T cells. Yellow fluorescence indicates restored YFP signal upon RSAD2–CMPK2 interaction (nYFP + cYFP reconstitution), and red fluorescence marks mitochondria. (scale bar, 10 µm). (E) Endogenous Co‑IP showing the interaction between RSAD2 and CMPK2 in BMDMs. (F) Coomassie‑brilliant‐blue staining of purified His‑RSAD2 and GST‑CMPK2 proteins. (G) GST‑pulldown assay demonstrating the direct interaction between His‑RSAD2 and GST‑CMPK2 in vitro. (H) Molecular docking simulation of RSAD2‐CMPK2 interaction; left, cartoon representation; right, stick model. (I) SPR sensorgrams of different concentrations of CMPK2 to RSAD2 immobilized on the CM5 sensor chip. The kinetic parameters were obtained by global fitting to a 1:1 Langmuir binding model, yielding a *K_D_* value of 526 nm. (J, K) Steady‐state binding curve of CMPK2 interaction with RSAD2. The *K_D_* value derived from affinity fitting was 450 nm. (L) Co‑IP of truncated CMPK2 fragments indicating the^1∼257aa^ domain responsible for RSAD2 binding. (M, N) Cross‐species sequence alignment of the RSAD2–CMPK2 binding domain. Highly conserved amino acid residues are shaded in red, and the protein–protein interaction sites are marked by green boxes.

Subcellular fractionation followed by Western blot showed increased mitochondrial localization of both RSAD2 and CMPK2 upon stimulation (Figure 6B and Figure S5A,B), and immunofluorescence confirmed RSAD2 translocation to mitochondria (Figure S5C). Protease K protection assays indicated that RSAD2 localizes primarily to the mitochondrial outer membrane, whereas CMPK2 resides in the intermembrane space/inner membrane (Figure 6C), suggesting their mitochondrial membrane proximity for potential interaction.

To elucidate the RSAD2‐CMPK2 interaction, we employed multiple approaches. Bimolecular fluorescence complementation (BiFC) (Figure S5D,H) showed yellow fluorescence in HEK‑293T cells co‐transfected with Rsad2‐nYFP and Cmpk2‐cYFP, predominantly localized to mitochondria (Figure 6D). Endogenous Co‐IP confirmed enhanced RSAD2‐CMPK2 binding in stimulated macrophages (Figure 6E and Figure S5F). GST pull‐down assays further validated direct interaction in vitro using purified proteins (Figure 6F,G). Schrödinger protein‐protein docking predicted binding between RSAD2 and CMPK2 residues Arg6 to Arg183 (Figure 6H and Figure S5E). SPR analysis demonstrated that purified CMPK2 bound to immobilized RSAD2 in a concentration‐dependent manner with a *K_D_* of approximately 500 nm (Figure 6I–K). Co‐IP with truncated CMPK2 mutants (Figure S5G) revealed that the N‐terminal 1∼257 amino acid region of CMPK2 is required for RSAD2 binding (Figure 6L). Cross‐species sequence alignment showed high conservation of the RSAD2‐CMPK2 interaction interface across human and mouse, (Figure 6M,N). Collectively, these results demonstrate that RSAD2 directly interacts with the N‐terminal domain of CMPK2_1∼257_ in stimulated macrophages.

### 2.6 Macrophage RSAD2 Dually Regulates CMPK2 Stability and Activity via Ubiquitination and Csnk2a2‐Mediated Phosphorylation

Database predictions revealed that CMPK2 is predicted to undergo both ubiquitination and phosphorylation modifications (Figure 7A), suggesting dual post‐translational regulation. Our prior findings demonstrate that RSAD2 enhances CMPK2 stability by suppressing its ubiquitination in stimulated macrophages. Protein stability assays revealed accelerated CMPK2 degradation and a shortened half‐life in *Rsad2* ^−/−^ macrophages (Figure 7B,C). Proteasomal inhibition with MG132, but not lysosomal inhibition with chloroquine, restored CMPK2 levels in *Rsad2* ^−/−^ macrophages (Figure 7D and Figure S6A), confirming degradation via the ubiquitin‐proteasome pathway. Co‐IP demonstrated that inflammatory stimulation reduced CMPK2 ubiquitination in *Rsad2* ^fl/fl^ macrophages, whereas *Rsad2* ^−/−^ macrophages exhibited restored ubiquitination levels (Figure 7E,F). Consistently, Co‐IP of CMPK2 from mitochondrial fractions confirmed that RSAD2 deficiency led to increased ubiquitination of mitochondrial CMPK2 upon stimulation (Figure S6B). Ubiquitin linkage analysis further established that CMPK2 undergoes K63‐linked (but not K48‐linked) ubiquitination (Figure S6C,D). Collectively, these results demonstrate that RSAD2 stabilizes CMPK2 by attenuating K63‑linked ubiquitination in activated macrophages (Figure 7G).

**FIGURE 7:**
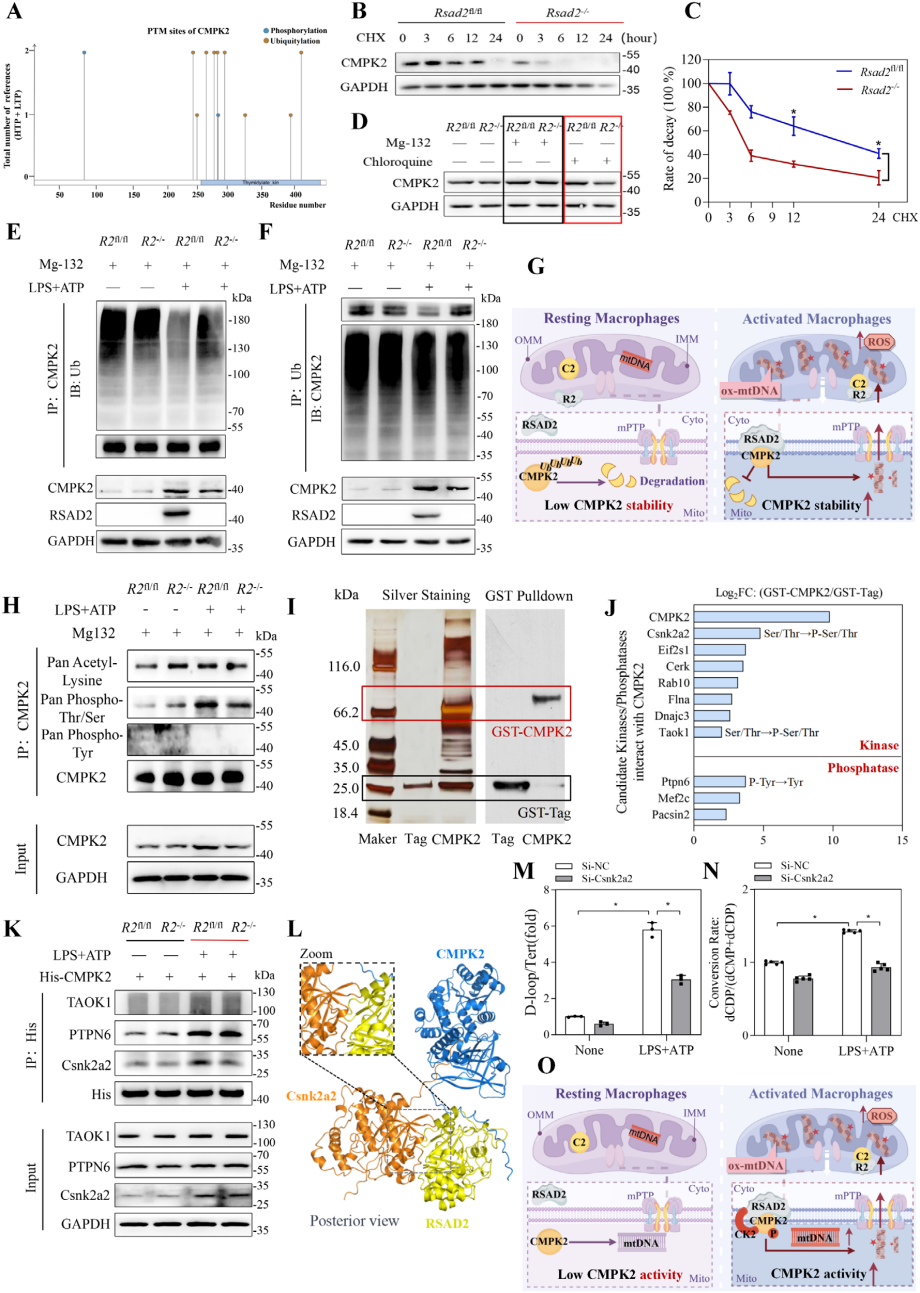
RSAD2 regulates dually phosphorylation and ubiquitination of CMPK2 to enhance mtDNA catalytic efficiency. (A) Prediction of post‐translational modification sites in amino acid residues of CMPK2. (B, C) Immunoblot analysis of CMPK2 protein stability in *Rsad2* ^fl/fl^ and *Rsad2* ^−/−^ BMDMs after CHX treatment at a time course gradient. (D) CMPK2 protein expression in *Rsad2* ^fl/fl^ and *Rsad2* ^−/−^ BMDMs treated with MG‐132 or chloroquine under LPS+ATP stimulation. MG‑132 or chloroquine was added 1 h before LPS+ATP stimulation. (E, F) Detection of CMPK2 ubiquitination levels by Co‐IP in *Rsad2* ^fl/fl^ and *Rsad2* ^−/−^ BMDMs before and after LPS + ATP stimulation. (G) Schematic model depicting RSAD2‐mediated regulation of CMPK2 ubiquitination in macrophages. (H) Representative immunoblots showing acetylation, serine/threonine phosphorylation, and tyrosine phosphorylation levels of CMPK2 in *Rsad2* ^fl/fl^ and *Rsad2* ^−/−^ BMDMs before and after LPS + ATP stimulation. (I) GST‐pulldown assay performed with bait proteins GST‑Tag and GST‑CMPK2 using lysates from LPS + ATP‐stimulated macrophages, followed by silver staining and GST‐immunoblot detection by SDS‑PAGE. (J) Heatmap of Log_2_ FC values for candidate kinases/phosphatases interacting with CMPK2 detected by mass spectrometry (GST‑CMPK2 vs GST‑Tag). (K) Co‑IP of CMPK2 with interacting proteins TAOK1, CSNK2A2, and PTPN6 in *Rsad2* ^fl/fl^ and *Rsad2* ^−/−^ BMDMs transfected with His‑CMPK2 before and after LPS + ATP stimulation. (L) Superimposed molecular model showing the binding mode among CSNK2A2, CMPK2, and RSAD2. (M) Quantification of mtDNA copy number in macrophages transfected with Si‐NC or Si‐Csnk2a2, with or without LPS+ATP treatment (n = 3). (N) Quantification of dCMP and dCDP levels by ELISA and calculation of CMPK2 catalytic conversion rate in BMDMs transfected with si‑NC or si‑Csnk2a2 before and after LPS + ATP stimulation. (O) Schematic model of RSAD2‐mediated regulation of CMPK2 phosphorylation in macrophages. Within‑group comparisons were analyzed by unpaired t tests; between‑group differences were determined by two‑way ANOVA followed by Tukey's post‑hoc test ^*^ *p* <0.05; NS, not significant.

Given that CMPK2 functions as a kinase, its catalytic activity, beyond mere protein abundance, is equally critical for biological function. Co‐IP with pan‐modification antibodies revealed that RSAD2 ablation specifically attenuated threonine phosphorylation of CMPK2 in stimulated macrophages, while tyrosine phosphorylation and acetylation remained unaltered (Figure 7H). As RSAD2 lacks enzymatic activity, we hypothesized it recruits kinases or phosphatases to modulate CMPK2 phosphorylation. GST pull‐down coupled with mass spectrometry identified CMPK2‐interacting proteins from LPS+ATP‐stimulated macrophages (Figure 7I and Figure S6E–G). Intersection with kinase/phosphatase databases prioritized seven kinases and three phosphatases (Figure 7J), with functional annotation highlighting Csnk2a2, TAOK1, and PTPN6 as top candidates. Co‐IP confirmed that RSAD2 ablation specifically reduced Csnk2a2‐CMPK2 binding without affecting PTPN6‐CMPK2, and no interaction was detected between TAOK1 and CMPK2 (Figure 7K). Molecular docking indicated that Csnk2a2 binds to residues Glu344‐Glu399 of CMPK2, a site distinct from the RSAD2‐binding interface (Figure S6H). Protein interaction modeling further suggested potential binding sites between RSAD2 and Csnk2a2 (Figure 7L and Figure S6I), supporting the hypothesis that RSAD2 facilitates Csnk2a2 recruitment to CMPK2. To directly validate that Csnk2a2 mediates CMPK2 phosphorylation, we knocked down Csnk2a2 in BMDMs. Additionally, siRNA‐mediated knockdown of *Csnk2a2* reduced both serine/threonine phosphorylation and protein expression of CMPK2 (Figure S6J–L), suggesting that phosphorylation may influence CMPK2 stability. For functional validation, siRNA‐mediated knockdown of *Csnk2a2* in BMDMs reduced CMPK2 catalytic efficiency regardless of stimulation, as evidenced by decreased mtDNA copy number (Figure 7M) and reduced dCDP conversion rate (Figure 7N and Figure S6M,N). Collectively, these results establish that RSAD2 recruits Csnk2a2 to phosphorylate and activate CMPK2 (Figure 7O). Together with its role in stabilizing CMPK2 protein, RSAD2 dually regulates CMPK2 via both ubiquitination and phosphorylation to enhance mtDNA synthesis and downstream inflammatory responses.

### 2.7 Macrophage RSAD2‐Noded mtDNA/cGAS Feedforward Loop Formation Regulates NLRP3 Inflammasome Activation

The results mentioned above demonstrate that RSAD2 acts as a key regulator of CMPK2, controlling its expression and function to ultimately influence mtDNA levels. It is known that mtDNA serves as a pivotal regulator of inflammatory responses, not only activating the NLRP3 inflammasome but also modulating the cGAS‐STING pathway. To investigate whether the cGAS‐STING‐IRF3 signaling axis transcriptionally upregulates *Rsad2* expression, we first treated mice with H‐151, a STING allosteric inhibitor (Figure 8A). H‐151 treatment suppressed cGAS‐STING pathway activation (Figure 8B,C) and reduced downstream IFN‐β (Figure 8D), IL‐1β (Figure 8E), and mtDNA copy number in F4/80^+^ macrophages (Figure 8F). Expression of RSAD2, CMPK2, NLRP3, and IL‐1β was also decreased (Figure 8B,C). To confirm transcriptional regulation, we used ChIP‐PCR. Potential IRF‐3 binding sites in the *Rsad2* promoter were predicted by JASPAR based on the interferon‐stimulated response element (ISRE) consensus sequence [30, 31] (Figure 8G). ChIP‐PCR revealed IRF‐3 binding to the *Rsad2* promoter at regions −742 to −511 bp (regions #3 and #4), whereas no binding was detected at other predicted sites (Figure 8H,I). Phenotypically, H‐151 treatment reduced inflammatory cell infiltration and accelerated wound closure (Figure 8J–M), normalized epidermal and dermal thickness, and reduced fibrosis (Figure 8N–Q). These results demonstrate that the cGAS‐STING pathway acts as an upstream signal orchestrating the RSAD2‐CMPK2‐mtDNA‐NLRP3 axis to modulate inflammatory responses and skin repair.

**FIGURE 8:**
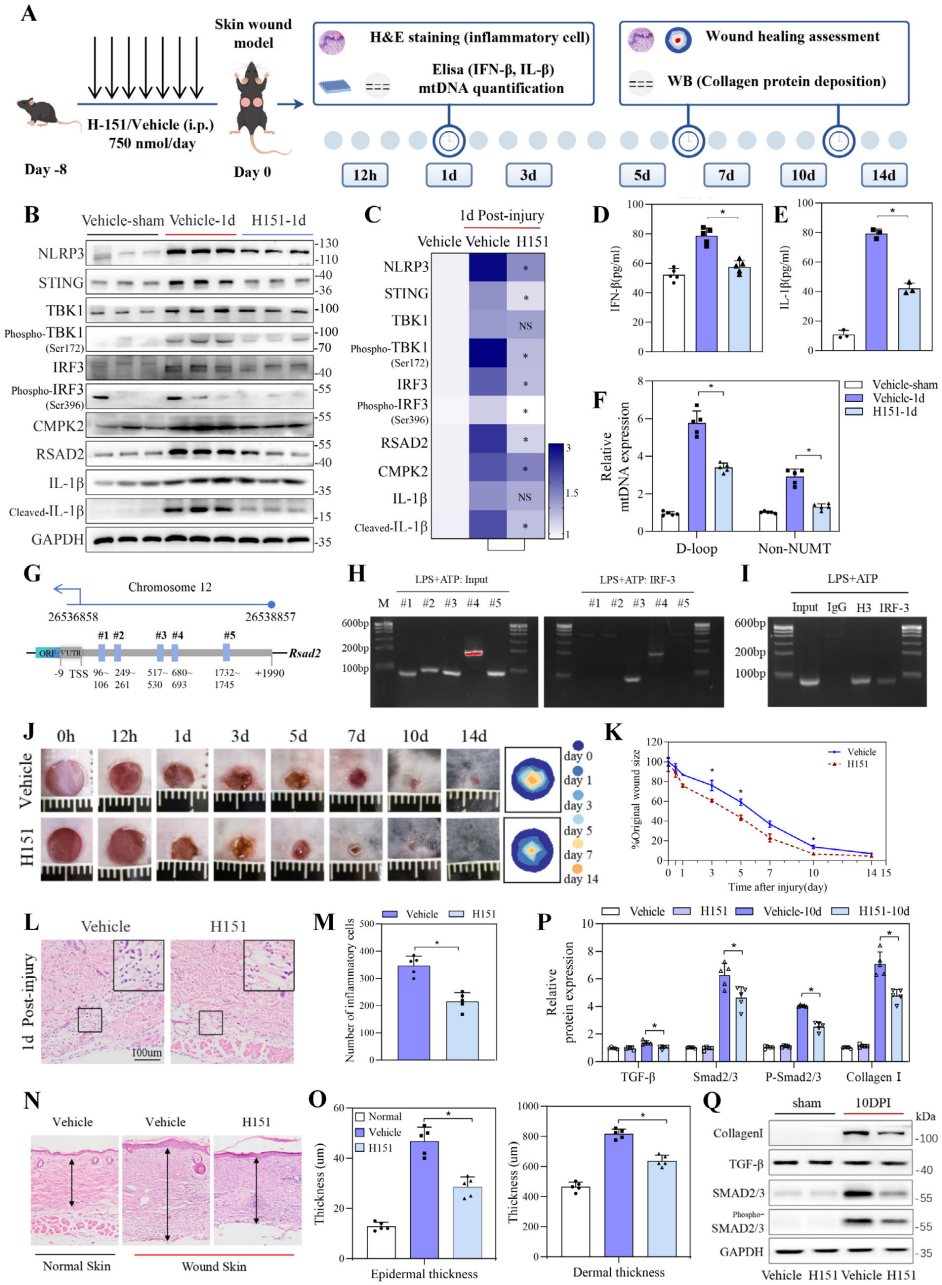
cGAS‐STING signaling regulates skin wound repair via transcriptional control of RSAD2. (A) Schematic diagram of the experimental design for STING depletion using H‐151 in a mouse skin wound model. (B, C) Representative immunoblots showing protein expression of the cGAS‐STING axis and the RSAD2‐CMPK2‐NLRP3 pathway in skin tissue of vehicle and H151‑treated mice under normal conditions and 1 day after injury, together with the quantitative heatmap. Expression values were normalized to the vehicle‑sham group. Within‑group comparisons were analyzed using unpaired t tests (n = 5). (D) Quantification of IFN‑β levels in skin tissue from vehicle and H151‐treated mice under normal and injured conditions. Within‑group comparisons were analyzed using unpaired t tests (n = 5). (E) Quantification of IL‐1β levels in skin wounds from Vehicle‐ and H‐151‐treated mice under normal and injured conditions. Within‑group comparisons were analyzed using unpaired t tests (n = 5). (F) qPCR analysis of mtDNA copy number in F4/80^+^ macrophages from vehicle and H151‑treated mice under normal and injured conditions. Within‑group comparisons were analyzed using unpaired t tests (n = 5). (G) Schematic representation of the Rsad2 promoter region showing predicted IRF‐3 binding sites. (H, I) ChIP‑PCR analysis showing the binding of transcription factor IRF‑3 to the *Rsad2* promoter in stimulated BMDMs. IgG served as a negative control and H3 as a positive control. (J, K) Representative images of skin wounds (J) and quantification of wound closure rates (K) in Vehicle‐ and H‐151‐treated mice during the wound healing process. Within‑group comparisons were analyzed using unpaired t tests (n = 3). (L, M) H&E staining of normal and 1‐day injured skin in vehicle and H151 group. (N, O) Quantification of epidermal thickness (N) and dermal scar thickness (O) in skin wounds from vehicle‐ and H‐151‐treated mice at 14 d post‐injury, compared to normal skin. (P, Q) Immunoblot and quantitative analysis of fibrosis‑related proteins in normal and 14 day injured skin from vehicle and H151 groups. Within‑group comparisons were analyzed using unpaired t tests (n = 5) ^*^ *p* <0.05; NS, not significant.

Having established cGAS‐STING as an upstream regulator of *Rsad2* transcription, we next investigated whether RSAD2‐regulated mtDNA feeds back to the cGAS‐STING pathway. We first characterized the binding properties of mtDNA to the two inflammasome complexes. Using a panel of primers targeting distinct mtDNA regions with balanced amplification efficiency in untreated BMDMs (Figure S7A), we performed affinity purification of mtDNA bound to cGAS and NLRP3 complexes (Figure 9A). In stimulated macrophages, IP of cGAS and NLRP3 complexes revealed that mtDNA fragments bound to cGAS across all regions, whereas binding to NLRP3 was D‐loop‐selective (Figure 9B,C). Time‐course analysis further showed that ox‐mtDNA engagement with cGAS peaked at 9 h, while engagement with NLRP3 peaked at 12 h post‐injury (Figure 9D,E), consistent with the temporal expression patterns of cGAS‐STING and NLRP3 proteins during skin repair (Figure S7B,C). Multiplex immunohistochemistry further validated a higher abundance of 8‐OHdG^+^/ASC^+^ macrophages than 8‐OHdG^+^/cGAS^+^ macrophages at 12 h (Figure S7D,E). Notably, RSAD2 ablation reduced cGAS‐bound ox‐mtDNA (Figure 9F). These results indicate that mtDNA simultaneously activates both cGAS‐STING and NLRP3 pathways during skin repair, with cGAS‐STING engagement preceding NLRP3 assembly in a temporally distinct manner. To test feedback regulation of RSAD2 on the cGAS‐STING pathway, we assessed pathway components in *Rsad2* ^fl/fl^ and *Rsad2* ^−/−^ skin tissues. RSAD2 deficiency suppressed injury‐induced activation of cGAS‐STING signaling (Figure 9G,H). Co‐IP assays further detected no interaction between RSAD2 and cGAS (Figure S7F), and molecular docking predicted a weak binding interface with limited contacts and a free energy of −18.18 kcal/mol (Figure S7G,H). In contrast, RSAD2 bound CMPK2 with much higher affinity (−52.5 kcal/mol) and extensive interfacial contacts. Thus, RSAD2 does not directly associate with cGAS; its feedback regulation of cGAS‐STING signaling is primarily mediated through the CMPK2–mtDNA axis rather than direct targeting of cGAS. Additionally, Rescue experiments with cGAMP (a STING agonist) partially restored p‐IRF3 nuclear translocation (Figure 9M) and cGAS‐STING pathway activation in *Rsad2* ^−/−^ macrophages (Figure 9I,K). Furthermore, cGAMP treatment upregulated CMPK2 and NLRP3 expression and enhanced inflammasome activation in *Rsad2* ^fl/fl^ macrophages, whereas RSAD2 deficiency reduced these responses; notably, cGAMP supplementation did not fully restore the defect in *Rsad2* ^−/−^ cells, highlighting a critical role for RSAD2 in mediating cGAS‐STING‐driven NLRP3 activation (Figure 9J,L). Together, these data establish RSAD2 as an essential amplifier of the mtDNA/cGAS feedforward loop that licenses coordinated NLRP3 inflammasome activation (Figure 9N).

**FIGURE 9:**
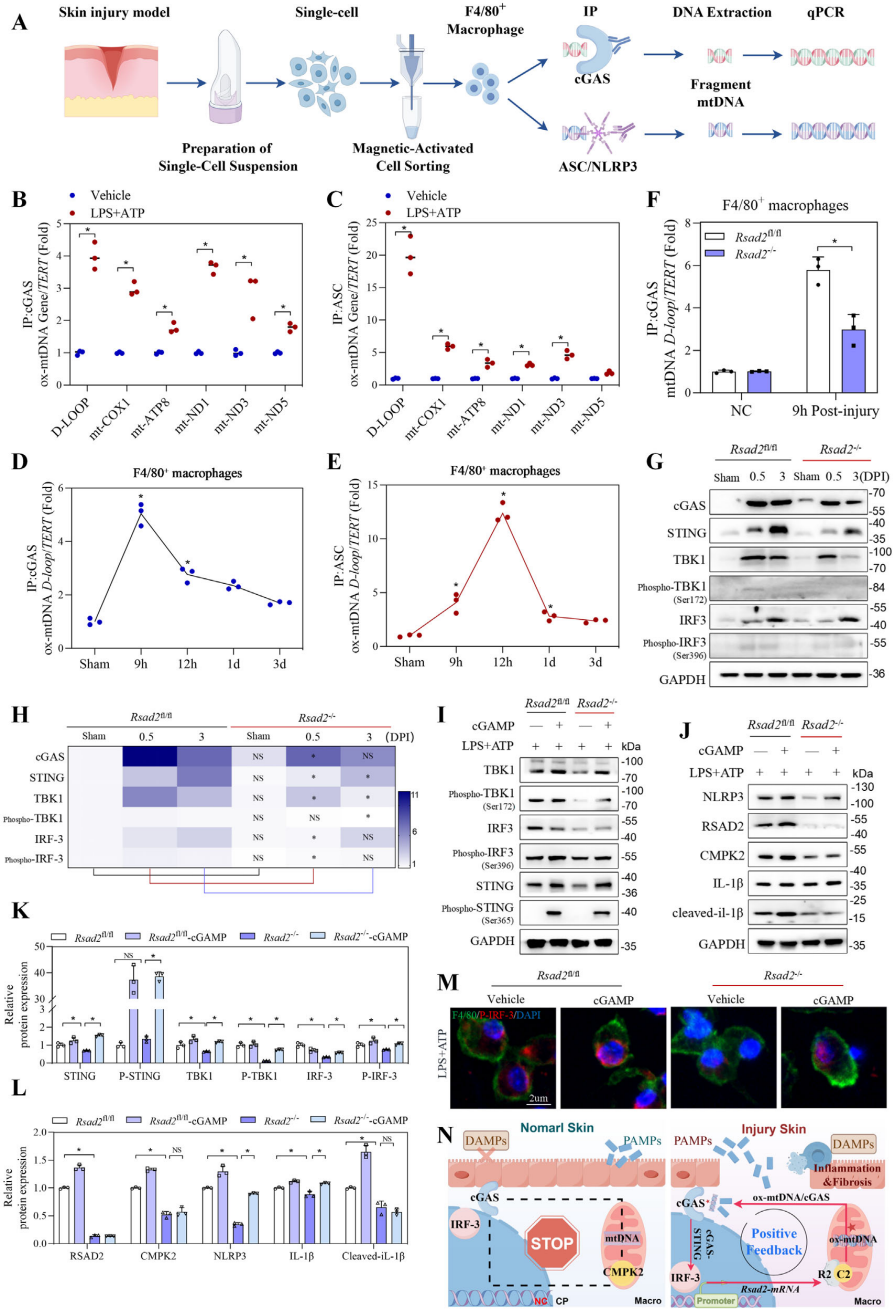
RSAD2 mediates an mtDNA/cGAS positive‑feedback loop and the temporal activation of the NLRP3 inflammasome after skin injury. (A) Schematic workflow for affinity purification of mtDNA bound to cGAS/NLRP3 inflammasome complexes in macrophages isolated from skin wounds. (B, C) qPCR analysis of ox‑mtDNA regions normalized to Tert within ASC and cGAS inflammasome complexes in BMDMs before and after stimulation. Values were normalized to the vehicle group. Within‑group comparisons were analyzed using unpaired t tests (n = 3). (D, E) Line graphs showing relative copy numbers of D‑loop derived ox‑mtDNA in cGAS and ASC inflammasome complexes isolated from F4/80^+^ cells in the wounded area at different time points after skin injury. Data were normalized to the sham group and analyzed by one‑way ANOVA followed by Tukey's post‑hoc test (n = 3). (F) Quantification of D‑loop‐derived ox‑mtDNA copy numbers within cGAS inflammasome complexes from F4/80^+^ cells of *Rsad2* ^fl/fl^ and *Rsad2* ^−/−^ mice under normal conditions and at 9 h after injury (n = 3; unpaired t test). (G, H) Representative immunoblots and quantitative heatmaps showing expression of cGAS‐STING pathway proteins in normal skin and 12 h and 3 d after injury. Data were normalized to the sham group and analyzed by two‑way ANOVA followed by Tukey's post‑hoc test (n = 5). (I, K) Western blot analysis (I) and quantification (K) of cGAS‐STING pathway proteins in *Rsad2* ^fl/fl^ and *Rsad2* ^−/−^ BMDMs treated with LPS+ATP, with or without cGAMP stimulation. Statistical analysis was performed using two‐way ANOVA followed by Tukey's post hoc test. (n = 3). (J, L) Western blot analysis (J) and quantification (L) of CMPK2 and NLRP3 inflammasome pathway proteins in *Rsad2* ^fl/fl^ and *Rsad2* ^−/−^ BMDMs treated with LPS+ATP, with or without cGAMP stimulation. Statistical analysis was performed using two‐way ANOVA followed by Tukey's post hoc test. (n = 3). (M) Representative immunofluorescence images of F4/80, p‐IRF‐3, and DAPI in *Rsad2* ^fl/fl^ and *Rsad2* ^−/−^ BMDMs treated with LPS+ATP, with or without cGAMP stimulation. Scale bar = 2 µm. (N) Schematic model illustrating the RSAD2‐mediated mtDNA/cGAS positive feedback loop following skin injury ^*^ *p* <0.05; NS, not significant.

### 2.8 Virtual Screening of RSAD2‐Targeting Small Molecules Accelerates Skin Wound Repair

Given the critical role of RSAD2 in regulating macrophage‐mediated inflammation and repair after skin injury, we performed structure‐based virtual screening against the mouse RSAD2 protein (Figure 10A) using the NeoraBio commercial compound library (35.1 K compounds). Based on docking scores (Figure S8A), two compounds, PDK1 inhibitor and PSB 06126, were selected as primary candidates. Molecular docking predicted a binding free energy of −13.6 kcal/mol for the PDK1 inhibitor with RSAD2, involving 4 hydrogen‐bonding interactions and 2 *π*–*π* interactions (Figure S8B,C). PSB 06126 showed a binding free energy of −12.9 kcal/mol with RSAD2, mediated by 5 hydrogen bonds and 1 *π*–*π* interaction (Figure 10B,C). Furthermore, L‐chicoric acid (LCA), previously identified from the MCE commercial compound library as an RSAD2‐targeting compound that improves pregnancy outcomes in SLE mice, was also included [32]. Thus, the PDK1 inhibitor, PSB 06126, and LCA were chosen for subsequent experimental validation.

**FIGURE 10:**
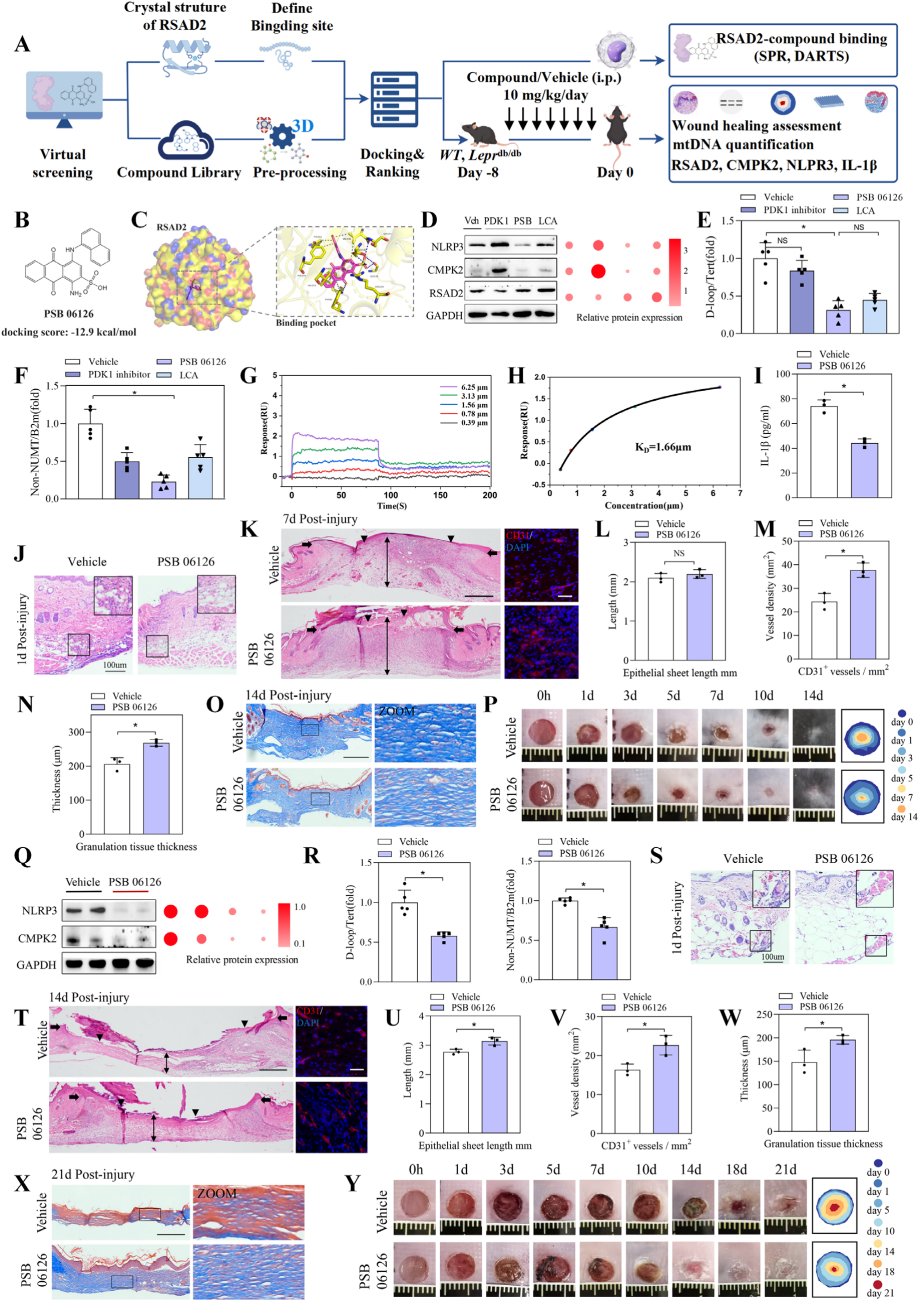
Virtual screening of an RSAD2‑targeted small‑molecule compound accelerates skin‑wound repair. (A) Workflow of virtual screening and experimental validation for RSAD2‑targeting compounds. (B) Chemical structure of PSB06126. (C) 3D binding model of PSB06126 with RSAD2 (left) and detailed analysis of the interaction interface (right). PSB06126 is shown as a rose‑colored stick representation; carbon atoms of mouse RSAD2 are shown in yellow, oxygen atoms in red, and hydrogen atoms in blue. (D) Schematic of drug administration. Mice received daily intraperitoneal injections of 10 mg/kg PDK1 inhibitor, PSB06126, LCA, or equivalent vehicle for 3 days before modelling. Western blot of NLRP3, CMPK2, and RSAD2 protein expression at 1 day after injury, normalized to the sham group. (E, F) Quantification of mtDNA levels by qPCR in F4/80^+^ cells magnetically isolated from wound areas 1 day after injury among different drug‑treatment groups. Data were analyzed by one‑way ANOVA followed by Tukey's post‑hoc test (n = 5). (G, H) SPR analysis of PSB 06126 binding to immobilized RSAD2. (G) Representative sensorgrams showing binding responses at increasing concentrations of PSB 06126. (H) Steady‐state binding curve of PSB 06126 interaction with RSAD2. The *K_D_* derived from affinity fitting was 1.66 um. (I) ELISA quantification of IL‐1β levels in skin wounds from vehicle‐ and PSB 06126‐treated mice at 1 d post‐injury (n = 3). (J) Representative H&E staining images and quantification of inflammatory‑cell infiltration in wounded areas 1 day after injury in vehicle and PSB06126 groups. (scale bar, 100 µm). (K) Representative H&E staining and CD31 immunofluorescence of granulation tissue in the vehicle and PSB 06126‐treated groups at day 7 post‐injury. Black triangles indicate the leading edge of the epithelial tongue; black arrows indicate the origin of the epithelial tongue (scale bar, 200 µm). (L–N) Quantification of epithelialization length, CD31^+^ vessel density, and granulation tissue thickness in the vehicle and PSB 06126‐treated groups at day 7 post‐injury. (O) Representative Masson's trichrome staining of granulation tissue 14 days after injury in vehicle and PSB06126 groups (scale bar, 200 µm). (P) Representative wound images and simulated healing curves of vehicle and PSB06126 ‑treated group. (Q) Immunoblot analysis of NLRP3 and CMPK2 protein expression in *Lepr* ^db/db^ mice 1 day after skin injury, normalized to the vehicle group. (R) Quantitative PCR analysis of mtDNA in F4/80^+^ cells from *Lepr* ^db/db^ mice treated with vehicle or PSB 06126 1 day after injury (n = 5; unpaired t test). (S) Representative H&E staining of wounded areas from *Lepr* ^db/db^ mice 1 day after injury (scale bar, 100 µm). (T) Representative H&E staining and CD31 immunofluorescence of granulation tissue in the vehicle and PSB 06126‐treated groups at day 7 post‐injury. Black triangles indicate the leading edge of the epithelial tongue; black arrows indicate the origin of the epithelial tongue (scale bar, 200 µm). (U–W) Quantification of epithelialization length, CD31^+^ vessel density, and granulation tissue thickness in the vehicle and PSB 06126‐treated groups at day 7 post‐injury. (X) Representative Masson's trichrome staining of granulation tissue from *Lepr* ^db/db^ mice 21 days after injury in vehicle and PSB06126 groups (scale bar, 200 µm). (Y) Representative wound images and simulated healing curves of vehicle and PSB06126 ‑treated *Lepr* ^db/db^ mice. Within‐group comparisons were analyzed using unpaired t tests, ^*^ *p* <0.05; NS, not significant.

To evaluate the anti‐inflammatory effects of these compounds after skin injury, we measured the expression of CMPK2 and the NLRP3 inflammasome, as well as mtDNA copy number in F4/80^+^ macrophages. Western blot revealed that while the PDK1 inhibitor reduced RSAD2 expression (Figure 10D), it markedly increased CMPK2 and NLRP3 levels, suggesting poor functional targeting of RSAD2 in vivo. In contrast, PSB 06126 and LCA did not significantly alter RSAD2 expression but substantially reduced CMPK2 and NLRP3 expression and decreased mtDNA copies in F4/80^+^ macrophages (Figure 10D–F). PSB 06126 exhibited the most pronounced anti‐inflammatory effect and was therefore selected as the lead compound for further study. SPR measurements further demonstrated a moderate binding affinity between PSB 06126 and RSAD2, with a *K_D_* of 1.66 µm (Figure 10G,H). DARTS assay further demonstrated that PSB 06126 protected RSAD2 from protease digestion in a concentration‑dependent manner, confirming direct interaction (Figure S8E).

In a mouse skin wound model, PSB 06126 treatment reduced inflammatory cell infiltration at 1 d (Figure 10I,J and Figure S8D). At day 7 post‑injury, PSB 06126‑treated mice exhibited significantly enhanced re‑epithelialization, increased CD31^+^ vessel density, and thicker granulation tissue (Figure 10K–N). Consistently, PSB 06126 treatment decreased fibrosis at 14 d (Figure 10O), and accelerated wound closure (Figure 10P). For safety assessment, PSB 06126 was administered to both uninjured and wounded mice (14 d post‑injury) under the same dosing regimen, with vehicle controls in each setting. In both sham and injured mice, PSB 06126 administration caused only mild changes in ALT levels, which remained within the normal reference range (22–133 U/L). No significant alterations were observed in other biochemical parameters, blood counts, or body weight. Histological examination of major organs revealed no abnormalities in either setting (Figure S8F–S). These data indicate that PSB 06126 is well‑tolerated at the therapeutic dose, regardless of injury status. To further assess efficacy in diabetic chronic wounds, we administered PSB 06126 to *Lepr* ^db/db^ mice. Consistent with previous results, PSB 06126 suppressed CMPK2 and NLRP3 expression (Figure 10Q), reduced mtDNA copies in F4/80^+^ macrophages (Figure 10R), attenuated inflammatory infiltration (Figure 10S and Figure S8T), and decreased IL‑1β levels (Figure S8U), At day 14 post‑injury, PSB 06126‑treated *Lepr* ^db/db^ mice exhibited significantly enhanced re‑epithelialization, increased CD31^+^ vessel density, and thicker granulation tissue compared to vehicle controls (Figure 10T–W), collectively indicating improved proliferative‑phase healing. Consistently, PSB 06126 treatment accelerated wound closure (Figure 10Y). Notably, Masson's trichrome staining at day 21 revealed more pronounced collagen deposition in the PSB 06126‑treated group compared to vehicle controls (Figure 10X). Consistent with the accelerated wound closure observed in PSB 06126‑treated mice, this observation likely reflects an advanced stage of the remodeling phase in the treatment group, whereas vehicle‑treated wounds had just completed re‑epithelialization with relatively low fibrosis at this time point. Together, these findings demonstrate that PSB 06126, a small molecule identified through virtual screening, effectively targets RSAD2 to suppress the CMPK2/mtDNA/NLRP3 axis, thereby alleviating inflammation, reducing fibrosis, and accelerating wound healing in both normal and diabetic mice. This establishes RSAD2 as a potential therapeutic target for improving scar formation and chronic non‑healing wounds associated with diabetes.

## 3 Discussion

This study integrates spatial transcriptomics with functional validation to reveal a novel immunometabolic role of macrophage RSAD2 as a crucial effector in skin repair after wounding. We discovered that RSAD2 is upregulated in macrophages and translocates to mitochondria upon injury. RSAD2 directly binds the N‐terminal domain (1∼256aa) of CMPK2 and dually regulates it through post‐translational modifications: it suppresses K63‐linked ubiquitination to enhance CMPK2 stability, and recruits Csnk2a2 to promote CMPK2 threonine phosphorylation, thereby boosting its catalytic activity and driving mtDNA synthesis. Newly synthesized mtDNA is converted to ox‐mtDNA via mtROS and released into the cytosol. Liberated ox‐mtDNA coordinately activates two inflammation‐related pathways: the mtDNA/cGAS feedback loop and NLRP3 inflammasome signaling.

In the mtDNA/cGAS feedback loop, cytosolic ox‑mtDNA is sensed by cGAS, initiating the cGAS‐STING‐IRF3 axis. Activated IRF3 then binds the *Rsad2* promoter region (−742 to −511 bp), establishing a self‐amplifying mtDNA/cGAS‐STING‐IRF3‐RSAD2 feedforward loop. In parallel, D‐loop‐derived ox‐mtDNA preferentially engages the NLRP3 inflammasome, triggering IL‑1β maturation and initiating inflammatory responses. Crucially, these two pathways exhibit spatial‑temporal uncoupling, with cGAS binding peaking at 9 h and NLRP3 binding peaking at 12 h during the inflammatory phase after skin injury. This dual cascade sustains the inflammatory response and modulates the inflammation‑to‑repair transition. Through virtual screening, we identified a small‑molecule compound, PSB 06126, that directly targets RSAD2 by binding to its CTP‑binding pocket, thereby suppressing CMPK2 expression and mtDNA production in macrophages after skin injury and attenuating wound‑induced inflammation. Overall, disruption of this circuit—through either RSAD2 inhibition or cGAS–STING blockade—significantly attenuates inflammation and accelerates healing. These findings position RSAD2 as a promising therapeutic target for immunometabolic intervention in inflammation‑related diseases.

Our findings advance the understanding of RSAD2 biology. While RSAD2 is traditionally recognized as a canonical ISGs with broad‐spectrum antiviral functions [18, 30], our study repositions RSAD2 as a central regulator of inflammatory responses. We systematically delineate a novel mechanism by which RSAD2 activates the NLRP3 inflammasome via the CMPK2/mtDNA axis in macrophage‑mediated inflammation. Consistent with previous reports showing that RSAD2 knockdown ameliorates aberrant immune responses in Sjögren's syndrome and psoriasis via NF‑κB suppression [28, 29, 33], we found that RSAD2 regulates NF‑κB activation. Moreover, independently of the priming signal, RSAD2 disrupts mitochondrial homeostasis to license NLRP3 inflammasome assembly. Significantly, we delineate the molecular basis of RSAD2‐CMPK2 cooperation in macrophages. Direct binding enables RSAD2 to stabilize CMPK2 by suppressing K63‑linked ubiquitination and to enhance its catalytic activity through Csnk2a2‑dependent threonine phosphorylation. Furthermore, our findings suggest that phosphorylation and ubiquitination of CMPK2 may not be entirely independent—knockdown of Csnk2a2 reduced both CMPK2 phosphorylation and protein levels [34, 35], raising the possibility that diminished phosphorylation may destabilize CMPK2 and promote its ubiquitin‑mediated degradation. Future studies are warranted to elucidate the potential interplay between these two post‑translational modifications. Together, this systematic exploration resolves mechanistic questions regarding functional RSAD2–CMPK2 interplay in inflammatory contexts [36].

At the level of RSAD2‐mediated mtDNA inflammatory regulation, our study uncovers distinct binding properties of ox‐mtDNA to cGAS or NLRP3 inflammasome complexes in macrophages. In the context of skin injury, NLRP3 preferentially binds to oxidized mtDNA fragments derived from the D‐loop region in macrophages, which aligns with previous reports [12, 37]. Nevertheless, the structural basis for this selectivity requires further investigation. In contrast, cGAS exhibits no regional binding preference, likely attributable to its broad nucleic acid recognition capacity and protein structural features [38, 39, 40]. Furthermore, we demonstrate a spatial‐temporal hierarchy in pathway activation post‐injury: ox‐mtDNA binding to cGAS precedes NLRP3 inflammasome engagement, revealing a kind of sequential organization of mtDNA‐mediated inflammatory events. Critically, we establish that RSAD2‐mediated mtDNA release activates the cGAS‐STING pathway, leading to nuclear translocation of IRF‐3 which transcriptionally upregulates *Rsad2* expression. This forms an mtDNA/cGAS feedforward circuit that advances NLRP3 inflammasome activation. Collectively, these findings delineate a crosstalk mechanism between cGAS‐STING signaling and NLRP3 inflammasome activation, deepen understanding of mitochondrial‐nuclear communication in inflammation, and provide new mechanistic frameworks for dissecting immune regulation in tissue repair.

While this study systematically deciphers the RSAD2‐centric regulatory axis in macrophage‐initiated inflammatory response, several limitations warrant consideration. First, although PSB 06126 was administered via intraperitoneal injection in this proof‑of‑concept study, future translational applications would benefit from optimized delivery strategies, such as topical application or incorporation into hydrogel‑based wound dressings, to minimize systemic exposure and enhance local bioavailability [41]. Second, we observed that RSAD2 overexpression induces subcutaneous fat thickening, a phenomenon aligning with reports linking RSAD2 to lipid droplet accumulation in systemic lupus erythematosus vasculitis [32] and its role as a fatty acid β‐oxidation regulator [42, 43]. This extends the functional spectrum of RSAD2 beyond innate immunity, implicating it as a potential molecular bridge linking inflammatory signaling to aberrant lipid metabolism. Thus, RSAD2 may exacerbate inflammation not only directly but also indirectly through metabolic reprogramming, a speculative yet promising axis for future research. Third, the mechanism underlying RSAD2 mitochondrial translocation remains undefined though we discovered its localization on the mitochondrial membrane. Our data support several non‐exclusive hypotheses: (I) Physical interaction with CMPK2, which contains intrinsic mitochondrial targeting sequence, may facilitate RSAD2 co‐trafficking; (II) Transporter proteins (e.g., heat shock proteins or mitochondrial signaling molecules) [44] could shuttle RSAD2 to mitochondria; (III) Upon macrophage activation, lipid droplet‐mitochondria tethering enables fatty acid transfer for bioenergetic support of inflammatory functions [45, 46]. This subcellular crosstalk might concurrently mediate RSAD2 relocation from lipid droplets. Definitive demonstration of these mechanisms requires further investigation.

Our study redefines the functional scope of RSAD2, transitioning its characterization from an antiviral factor to a central immunometabolic regulator. Our findings carry multifaceted significance. Mechanistically, we establish a new model of sequential coordination between an mtDNA/cGAS feedforward loop and NLRP3 effector pathway, unveiling dynamic communication between mitochondria and nucleus. Additionally, the RSAD2‐CMPK2 axis emerges as a druggable target, with RSAD2‐specific inhibitors holding promise for intervening inflammatory illnesses. Finally, the conserved nature of this mechanism warrants exploration of RSAD2 in other inflammatory and metabolic disorders.

## 4 Materials and Methods

### 4.1 Mice

*Rsad2* ^fl/fl,^ *Lyz2*‐iCre mice, *Nlrp3* ^−/−^ and *Lepr* ^db/db^(Strain No. T019755, T003822, T010873 and T002407, respectively) were obtained from GemPharmatech (Nanjing, China) and maintained under SPF conditions in the Laboratory Animal Center of China Medical University. *Rsad2* ^fl/fl^ mice were used as controls (*Rsad2* ^+/+^). *Rsad2* ^fl/fl^ *Lyz2*‐iCre mice represent macrophage‐specific knockout mice (*Rsad2* ^−/−^). *Lepr* ^db/db^ mice were used to model diabetic chronic wounds. The STING inhibitor H‐151 (MCE, HY‐112693) was administered via intraperitoneal injection to suppress STING signaling. Mice received daily injections of H‐151 (750 nmol in 200 µL) PSB 06126 (10 mg/kg), or an equal volume of vehicle for 7 consecutive days prior to modeling. An additional dose was administered immediately after surgery, followed by continued once‐daily injections until tissue collection.

### 4.2 Skin Wound Model

The skin wound model was established as previously described. Briefly, mice were anesthetized using isoflurane inhalation (2.5% at a flow rate of 200 mL/min), and the dorsal fur was removed. Under aseptic conditions, a full‐thickness excisional skin wound was created with a 6 mm disposable biopsy punch, positioned 1 cm lateral to the midline on the upper back. Control mice received anesthesia and hair removal but did not undergo skin injury. All animals were housed individually post‐surgery with ad libitum access to food and water. Wounds were monitored daily and kept clean to prevent infection. All experimental procedures were conducted in accordance with institutional guidelines and approved by the Animal Ethics Committee of China Medical University (Approval No. CMU2023898).

### 4.3 Human Skin Samples Collection and Medical Ethics

This study was approved by the Ethics Committee of China Medical University (Approval No. 18, 2022). Human skin wound samples and adjacent normal tissues were obtained from forensic autopsy cases with a post‑injury interval of ≤3 days. All samples were collected from cadavers preserved at 4°C or frozen within 72 h post‑mortem, with no documented history of diseases or medications that affect skin wound repair (Table S1).

### 4.4 Single‐Cell Suspension Preparation

Mice were euthanized by CO_2_ asphyxiation at indicated time points. Skin tissues were collected, minced, and digested using a Gentle Tissue Dissociation Kit (RWD, #H‐601‐03443) according to the manufacturer's instructions. Single‐cell suspensions were obtained using an SCT‐100 automated dissociator, filtered through a 100 µm strainer, and centrifuged. Cell pellets were resuspended in PBS and treated with a debris removal reagent, followed by centrifugation at 3000×g for 10 min at 4°C. Supernatants were discarded.

### 4.5 Magnetic Sorting of Macrophages From Skin Wounds

Single‐cell suspensions were prepared from skin wound tissues as described above. Macrophages were isolated by magnetic‐activated cell sorting using Anti‐F4/80 MicroBeads UltraPure (#130‐110‐443, Miltenyi Biotec), Anti‐Ly‐6G MicroBeads UltraPure (#130‐120‐337, Miltenyi Biotec), CD3ε MicroBead Kit (#130‐094‐973, Miltenyi Biotec). Briefly, cells were incubated with the microbeads for 15 min at 4°C, washed, and then applied to MS Columns (#130‐042‐201, Miltenyi Biotec) placed in a magnetic field. After washing, the column was removed from the magnetic field, and labeled cells were eluted. Purified macrophages were collected for subsequent analyses.

### 4.6 Mouse Inflammatory Cytokine Antibody Array

Inflammatory cytokine expression in skin tissues was assessed using a mouse inflammation antibody array (#422247157, RayBiotech). Briefly, tissue protein samples were diluted to 500 µg/mL. The membrane was blocked and then incubated with samples overnight at 4°C. After washing, HRP‐conjugated streptavidin and a detection antibody mixture were added sequentially. Signals were developed by chemiluminescence, and cytokine expression levels were analyzed based on the predefined spot layout.

### 4.7 Mitochondrial DNA Quantification

Total DNA was extracted from SMNCs or BMDMs using the FastPure DNA Isolation Mini Kit (#DC112, Vazyme) according to the manufacturer's instructions. DNA concentration and purity were measured spectrophotometrically, and samples were normalized to 20 ng/µL. Quantitative PCR was performed using a LightCycler 480 system with specific primers (Table S3). The relative mitochondrial DNA copy number was calculated using the 2–^ΔΔCt^ method and expressed as the ratio of *D‐loop* to *Tert* and *non‐NUMT* to *B2m*, as previously research described.

### 4.8 mtDNA Comet Assay in Macrophages

BMDMs were cultured overnight prior to experiments. Mitochondria were isolated from the cytosolic fraction as described above. The mtDNA comet assay was performed using a Comet Assay Kit (#C2041S, Beyotime). Briefly, a two‐layer agarose gel was prepared: the first layer consisted of 1% normal melting point agarose, and the second layer contained isolated mitochondria embedded in 0.7% low melting point agarose. After solidification, slides were lysed at 4°C for 1–2 h, equilibrated in comet assay buffer, and electrophoresed at 25 V for 20 min on ice in the dark. Slides were then neutralized, stained with propidium iodide, and visualized under a confocal microscope.

### 4.9 Protease K Protection Assay

Mitochondria were isolated from LPS+ATP‐treated BMDMs as previously described. The mitochondrial pellet was divided into four aliquots and resuspended under the following conditions: (I) untreated control in mitochondrial storage buffer; (II) 100 ng/mL protease K in storage buffer; (III) 100 ng/mL protease K in hypotonic buffer (20 mm HEPES, pH 7.4); and (IV) 100 ng/mL protease K in storage buffer containing 0.1% Triton X‐100. After incubation on ice for 10 min, samples were denatured in loading buffer and analyzed by western blotting. The submitochondrial localization of RSAD2 and CMPK2 was determined using markers for outer membrane TOM20 (#11802‐1‐AP, Proteintech), intermembrane space Mia40/CHCHD4 (#21090‐1‐AP, Proteintech), and matrix HSP60 (#15282‐1‐AP, Proteintech).

### 4.10 Bimolecular Fluorescence Complementation (BiFC) Assay

Protein interaction between RSAD2 and CMPK2 was visualized using a BiFC assay. HEK 293T cells were seeded in confocal dishes and co‐transfected with constructed plasmids expressing Rsad2‐YN155 and Cmpk2‐YC155 fusion proteins, along with corresponding empty vectors as negative controls, using Lipofectamine 3000. After 8 h, the medium was replaced with fresh culture medium, and cells were further incubated for 24 h. Subsequently, cells were stained with Hoechst 33342 and MitoTracker Red to label nuclei and mitochondria, respectively, followed by imaging under a confocal microscope to detect BiFC signals.

### 4.11 Protein Purification and GST Pull‐Down Assay

The coding sequences of *Rsad2* and *Cmpk2* were cloned into pET‐22b and pGEX‐6P‐1 vectors, respectively. The constructs were transformed into E. coli BL21(DE3) competent cells. His‐tagged RSAD2 protein was expressed following induction with IPTG and purified using Ni‐NTA affinity chromatography with elution using 250 mm imidazole. GST‐tagged CMPK2 was purified using glutathione Sepharose resin and eluted with 20 mm reduced glutathione. Protein purity and molecular weight were confirmed by SDS‐PAGE. For GST pull‐down assays, glutathione Sepharose beads were incubated with either GST or GST‐CMPK2 fusion protein at 4°C for 1 h. After washing, the beads were incubated with His‐RSAD2 protein overnight at 4°C. The beads were washed extensively to remove unbound proteins, and bound complexes were eluted and analyzed by SDS‐PAGE and western blotting using an anti‐GST monoclonal antibody (#M20007S, Abmart).

### 4.12 Surface Plasmon Resonance (SPR)

SPR experiments were performed on a Biacore 1K instrument using a Series S Sensor Chip CM5 (#10344853, Cytiva). The running buffer (10× PBS, 0.05% Tween‐20, and 1% DMSO) was degassed prior to use. For amine‐coupling immobilization (#BR100050, Cytiva), the CM5 surface was activated with a 1:1 mixture of 0.4 m EDC and 0.1 m NHS at 10 µL/min, followed by injection of the target protein in 10 mm sodium acetate to an immobilization level of 3000 RU. Residual activated groups were blocked with ethanolamine at 10 µL/min. Small‐molecule analytes were prepared in PBST (1% DMSO) and serially diluted. Binding kinetics were collected at 25°C under multicycle conditions after three startup cycles and blank injections. Sensorgrams were double‐referenced, and KON, KOFF, and KD values were obtained in Biacore Evaluation Software (Cytiva, Sweden) by global fitting to a 1:1 Langmuir binding model. The values were smoothened by averaging values per three points.

## Author Contributions

**Shukui Du**: investigation, validation. **Shuyang Mu**: data curation. **Jinnong Yang**: formal analysis. **Chongchong Xu**: data curation. **Haomiao Yuan**: investigation, validation, writing – original draft, visualization, methodology, writing – review and editing. **Yijun Wang**: validation. **Jiayang Xiao**: validation. **Rui Zhao**: conceptualization, funding acquisition, supervision. **Jingkai Sun**: formal analysis. **Anran Qu**: software. **Linlin Wang**: investigation. **Xinjie Li**: investigation. **Hengxu Yan**: software. **Dawei Guan**: conceptualization, supervision, funding acquisition, methodology. **Tiegang Li**: conceptualization, funding acquisition, supervision. **Chunfei Li**: investigation, validation. **Fuyuan Zhang**: investigation.

## Funding

This study was financially supported by the National Natural Science Foundation of China (Grant Nos. 82271926, 81871529)

## Ethics Statement

All animal experiments were approved by the Animal Experiment Committee of China Medical University (Approval No. CMU2023898) and conducted in accordance with the National Institutes of Health Guide for the Care and Use of Laboratory Animals. Human tissue samples were obtained with informed consent and used under protocols approved by the Ethics Committee of China Medical University (Approval No. 18 [2022]), in compliance with the principles of the Declaration of Helsinki.

## Conflicts of Interest

The authors declare no conflicts of interest.

## Supporting information




**Supporting File**: advs77416‐sup‐0001‐SuppMat.docx.

## Data Availability

The spatial transcriptomics data generated in this study have been deposited in the Sequence Read Archive (SRA) under accession number PRJNA1510020. The human dataset (GSE241132) analyzed during the current study is available in the Gene Expression Omnibus (GEO) repository. All other data supporting the findings of this study are available from the corresponding author upon reasonable request.
