# Supplementary material for: Macrophage RSAD2 Couples mtDNA Synthesis With a Self‐Amplifying Inflammatory Circuit to Orchestrate Tissue Repair
Source: Adv Sci (Weinh). 2026 Aug 29:e77416. Online ahead of print. doi: 10.1002/advs.77416 (PMC13525419; doi:10.1002/advs.77416)
Supplement: Supplementary file 1 [file ADVS-9999-e77416-s001.docx]

**Supplementary Figures**

**Figure S1. Spatial transcriptomic profiling of skin‑wound repair reveals spatiotemporal expression of RSAD2**

(A) Cell type annotation of spatial transcriptomic spots based on canonical markers and the CellMarker database.

(B) Dynamic changes in the proportions of major cell types in the wound area at indicated time points post-injury. Colors represent distinct cell lineages.

(C) GO enrichment analysis of differentially expressed genes in the wound area during skin wound repair.

(D) Expression of ligands (left) and their cognate receptors (right) across different cell types during wound healing, depicted as heatmaps. Colors indicate cell types.

(E) KEGG pathway enrichment of  RSAD2⁺ SPOTs identified during skin wound and repair.

(F) Representative gating strategy for multicolor flow cytometry analysis of skin mononuclear cells.

(G) t‑SNE analysis of the proportion of  RSAD2⁺ cells among CD86^+^, CD163^+^, and CD206^+^ macrophages (F4/80^+^) isolated from skin wounds at day 3 post‑injury by MACS. Each dot represents a single cell, colored by marker expression.

(H) Annotation of immune cell subsets (myeloid cells, lymphocytes, and mast cells) in human skin based on the GSE241132 dataset, using canonical markers.

**Figure S2. RSAD2 promotes inflammatory cell infiltration in skin wounds.**

(A, B) Genotyping of *Rsad2-Loxp* and *Lyz2*-iCre fragments in *Rsad2*^fl/fl^ *Lyz2*-iCre mice by PCR.

(C) Immunofluorescence validation of RSAD2 deletion in macrophages from *Rsad2*^fl/fl^ *Lyz2*-iCre mice. Scale bar = 10 μm.

(D, E) Validation of AAV9-CD68-Rsad2+-GFP injection efficiency and CD68 promoter specificity in mouse skin. Scale bar = 200 μm.

(F, G, H) Representative contour plots showing the purity of neutrophils (CD11b⁺Ly6G⁺), macrophages (CD11b⁺F4/80⁺), and T cells (CD3ε⁺) isolated from skin wounds by magnetic‑activated cell sorting (MACS).

(I) RT-qPCR analysis of *Rsad2* mRNA expression in neutrophils, macrophages, and T cells isolated from skin wounds of *Rsad2*^fl/fl^ and *Rsad2*^⁻/⁻^ mice at 1 day post‑injury.

(J, K) ELISA quantification of IL-6 and IL-1β levels in normal skin and wound areas at  1 d after injury in *Rsad2*^fl/fl^ and *Rsad2*^-/-^ mice, n=5.

(L, M) ELISA quantification of IL‑6 and IL‑1β levels in normal skin and wound areas at  1 d after injury in AAV-Vector and AAV-Rsad2^+^ mice, n=5.

(N, O, P, Q, R) Representative H&E, toluidine blue MPO, and F4/80 IHC images showing inflammatory cell infiltration in normal skin and wound areas at 12 h and 3 d after injury in AAV-Vector and AAV-Rsad2^+^ mice, along with quantification of total inflammatory cells, neutrophils, macrophages, and mast cells in the indicated groups, n = 5.

Within-group comparisons were analyzed using unpaired t tests. **p* < 0.05; NS, not significant.

**Figure S3. RSAD2‑mediated inflammatory responses affect fibrotic wound repair after skin injury**

(A) Representative wound images of *Rsad2*^fl/fl^, *Rsad2*^-/-^, and AAV9‑Rsad2⁺ mice during skin wound repair.

(B) Line graph showing wound‑closure rates of *Rsad2*^fl/fl^, *Rsad2*^-/-^, and AAV9‑Rsad2⁺ mice throughout the healing period.

(C) Representative H&E staining and CD31 immunofluorescence of granulation tissue in *Rsad2*^fl/fl^, *Rsad2*^-/-^, and AAV9‑Rsad2⁺  groups at day 7 post-injury. Black triangles indicate the leading edge of the epithelial tongue; black arrows indicate the origin of the epithelial tongue (scale bar, 200 μm).

(D, E, F) Quantification of epithelialization length , CD31⁺ vessel density , and granulation tissue thickness in the *Rsad2*^fl/fl^, *Rsad2*^-/-^, and AAV9‑Rsad2⁺ groups at day 7 post-injury.

(G) Representative Masson’s trichrome staining and collagen I immunofluorescence of scar tissue in *Rsad2*^fl/fl^, *Rsad2*^-/-^, and AAV9‑Rsad2⁺ mice groups at day 10 post-injury (scale bar, 200 μm).

(H) Immunoblot analysis of fibrosis‑related proteins in the skin tissue of *Rsad2*^fl/fl^ and *Rsad2*^-/-^ mice during skin wound repair.

(I) Representative H&E images of normal and 14‑day‑injured skin from *Rsad2*^fl/fl^, *Rsad2*^-/-^, and AAV9‑Rsad2⁺ mice (scale bar, 200 μm).

(J, K) Quantification of epidermal and dermal scar thickness in normal and 14‑day‑injured skin of  *Rsad2*^fl/fl^, *Rsad2*^-/-^, and AAV9‑Rsad2⁺ mice.

**p* < 0.05; NS, not significant.

**Figure S4 RSAD2 manipulates NLRP3 inflammasome activation in macrophages through the CMPK2/mtDNA pathway**

(A) KEGG pathway enrichment analysis of differentially expressed genes in Rsad2⁺ macrophage spots. **p* < 0.0025.

(B) Genotyping of *WT* and *Nlrp3* fragments in *Nlrp3*^⁻/⁻^ mice by PCR.

(C) Western blot analysis of NLRP3, RSAD2, IL-1β, and cleaved IL-1β in skin wounds from AAV-Vector, AAV-Rsad2⁺, AAV-Vector/*Nlrp3*^⁻/⁻^, and AAV-Rsad2⁺/*Nlrp3*^⁻/⁻^ mice at 1 d post-injury.

(D, E) Western blot analysis and quantification of NF-κB and phospho-NF-κB in *Rsad2*^fl/fl^ and *Rsad2*^-/-^ BMDMs before and after LPS+ATP stimulation.

(F) Representative immunoblots of NLRP3, pro‑caspase‑1, cleaved caspase‑1, pro‑IL‑1β, cleaved IL‑1β, and GAPDH in cell lysates of *Rsad2*^fl/fl^ and *Rsad2*^⁻/⁻^ BMDMs treated with LPS alone, ATP alone, or LPS+ATP.

(G, H) ELISA quantification of IL‑1β and IL‑18 in culture supernatants of *Rsad2*^fl/fl^ and *Rsad2*^⁻/⁻^ BMDMs treated with LPS alone, ATP alone, or LPS+ATP, (n = 3).

(I) Representative confocal images of ASC speck formation in *Rsad2*^fl/fl^ and *Rsad2*^⁻/⁻^ BMDMs upon LPS+ATP stimulation. Nuclei were counterstained with DAPI.

(J) Quantification of ASC speck‑positive cells in *Rsad2*^fl/fl^ and *Rsad2*^⁻/⁻^ BMDMs upon LPS+ATP stimulation, n = 3.

(K, L) Western blot analysis and quantification of NLRP3, Cathepsin B, Cathepsin D, and RSAD2 in *Rsad2*^fl/fl^ and *Rsad2*^⁻/⁻^ BMDMs before and after LPS+ATP stimulation (n = 3)

(M-O) Flow-cytometric quantification of DQ-BSA fluorescence (M), intracellular Ca^2+^ levels (N), and ROS production (O) in *Rsad2*^fl/fl^ and *Rsad2*^-/-^ BMDMs before and after LPS+ATP stimulation (n = 3).

(P) Representative flow cytometry plots of JC-1 staining in *Rsad2*^fl/fl^ and *Rsad2*^⁻/⁻^ BMDMs with or without indicated treatments (n = 3).

(Q, R) ELISA quantification of dCMP and dCDP in *Rsad2*^fl/fl^ and *Rsad2*^⁻/⁻^ BMDMs with or without LPS+ATP treatment. (n = 5).

(S, T) qPCR analysis of the D-loop copy number (normalized to Tert) and non-NUMT copy number (normalized to B2m) in F4/80^+^ macrophages (isolated by magnetic sorting) from *Rsad2*^fl/fl^ and *Rsad2*^-/-^ mice at indicated time points (n = 5).

(U) Immunofluorescence staining of ASC (green) and EdU (red) in *Rsad2*^fl/fl^ and *Rsad2*^-/-^ BMDMs with or without indicated treatments. Scale bar = 2 μm.

(V) Quantification of closed MPTPs in *Rsad2*^fl/fl^ and *Rsad2*^-/-^ BMDMs with or without LPS+ATP treatments.

(W) Spatial transcriptomic map showing the spatial distribution of *Rsad2* and *Cmpk2* expression during skin wound repair (scale bar, 400 μm).

(X, Y) Representative immunoblots of RSAD2 and CMPK2 expression during the wound repair process and correlation analysis of their levels.

Within-group comparisons were analyzed using unpaired t tests; between‑group differences were determined by two-way ANOVA followed by Tukey’s post‑hoc test. **p* < 0.05; NS, not significant.

**Figure S5. RSAD2 translocates to mitochondria and interacts with CMPK2^1~257aa^**

(A, B) Quantification of RSAD2 and CMPK2 protein levels in mitochondrial and cytosolic fractions from macrophages with or without LPS+ATP treatment, (n = 5).

(C) Representative immunofluorescence images showing co‑localization of RSAD2 (green) with Mito‑Tracker Red (red) in macrophages with or without LPS+ATP treatment. Scale bar = 5 μm.

(D) Schematic diagram illustrating the principle of the Bimolecular Fluorescence Complementation (BiFC) assay for detecting protein–protein interactions.

(E) Predicted binding interface between RSAD2 and CMPK2 by molecular docking.

(F) Co‑IP of endogenous RSAD2 and CMPK2 in macrophages with or without LPS+ATP treatment.

(G) Schematic representation of CMPK2 functional domains and truncation constructs used for mapping the RSAD2‑interacting region.

(H) SDS‑PAGE analysis validating BiFC plasmid transfection efficiency in HEK‑293T cells. Comparisons within each group were performed using unpaired Student's t‑test, and comparisons between groups were analyzed by two‑way ANOVA followed by Tukey's post hoc test.

**Figure S6 RSAD2 regulates dually phosphorylation and ubiquitination of CMPK2 to enhance mtDNA catalytic efficiency.**

(A) Quantification of CMPK2 protein expression in *Rsad2*^fl/fl^ or *Rsad2*^-/-^ BMDMs treated with MG-132 or chloroquine under LPS+ATP stimulation, MG‑132 (10 μM) or chloroquine (10 μM) was added 1 h before LPS (500 ng/mL, 4 h) and ATP (5 mM, 1 h) stimulation.

(B) Co-immunoprecipitation analysis of ubiquitination of CMPK2 in mitochondrial fractions (Mito) isolated from *Rsad2*^fl/fl^ and *Rsad2*^-/-^ BMDMs with or without LPS+ATP treatment.

(C, D) Co-immunoprecipitation analysis of K48- and K63-linked ubiquitination levels of CMPK2 in *Rsad2*^fl/fl^ and *Rsad2*^-/-^ BMDMs with or without LPS+ATP treatment.

(E) Venn diagram showing the number of macrophage proteins identified by mass spectrometry interacting with GST-Tag (control) or GST-CMPK2.

(F) Venn diagram illustrating candidate CMPK2-interacting proteins, proteins with kinase activity, and proteins with phosphatase activity.

(G) Pfam domain analysis of proteins specifically identified in the GST-CMPK2 pull-down group.

(H) Predicted interaction interface between CSNK2A2 and CMPK2. Yellow dashed lines represent hydrogen bonds, and purple dashed lines represent salt bridges.

(I) Predicted interaction interface between CSNK2A2 and RSAD2. Yellow dashed lines represent hydrogen bonds, and purple dashed lines represent salt bridges.

(J) Co-immunoprecipitation analysis of serine/threonine phosphorylation of CMPK2 in BMDMs transfected with Si-NC or Si-Csnk2a2 with LPS+ATP treatment.

(K, L) Representative immunoblots and quantification of CMPK2 protein expression in BMDMs transfected with Si-NC or Si-Csnk2a2, with or without LPS+ATP treatment (n = 3).

(M, N) ELISA quantification of dCMP and dCDP levels in BMDMs transfected with Si-NC or Si-Csnk2a2, with or without LPS+ATP treatment (n = 3).

Within‑group comparisons were analyzed by unpaired t tests; between‑group differences were determined by two‑way ANOVA followed by Tukey’s post‑hoc test.

**p* < 0.05; NS, not significant.

**Figure S7. RSAD2 mediates mtDNA/cGAS positive‑feedback loop and the temporal activation of the NLRP3 inflammasome after skin injury**
(A) qPCR analysis of mtDNA copy numbers targeting various mitochondrial regions in untreated BMDMs, normalized to Tert and expressed relative to the D-LOOP region. Statistical analysis was performed using one-way ANOVA followed by Tukey's post hoc test. (n=3).

(B, C) Western blot analysis (B) and quantification heatmap (C) of cGAS-STING pathway and NLRP3 protein expression in mouse skin under normal conditions and at 9 h, 12 h, 1 d, and 3 d post-injury. Expression levels were normalized to the sham group (set as 1). Statistical analysis was performed using one-way ANOVA followed by Tukey's post hoc test. (n=3).

(D,  E) Representative multiplex immunohistochemistry images of F4/80, 8‑OHdG, cGAS, and ASC in wound areas at 9 h and 12 h after injury (scale bar, 100 μm), and quantification of ASC- & 8‑OHdG‑positive and cGAS- & 8‑OHdG‑positive cells within F4/80⁺ fields. Within‑group comparisons were analyzed using unpaired t tests (n = 3).

(F)  Endogenous Co‑IP analysis of RSAD2 and cGAS in BMDMs.

(G, H)  Predicted interaction interface between RSAD2 and cGAS. Yellow dashed lines represent hydrogen bonds, and purple dashed lines represent salt bridges.

**Figure S8. Virtual screening of an RSAD2‑targeted small‑molecule compound accelerates skin‑wound repair**
(A) Top‑10 scoring compounds identified from virtual screening targeting RSAD2.

(B) Chemical structure of PDK1 inhibitor.

(C) Three-dimensional binding model of PDK1 inhibitor with RSAD2 (left) and detailed view of the interaction interface (right). PDK1 inhibitor is shown as rose-colored sticks. The carbon backbone of mouse RSAD2 is depicted in yellow, oxygen atoms in red, and hydrogen atoms in blue.

(D) Quantification of inflammatory cells in skin wounds from WT mice at 1 d post-injury, treated with vehicle or PSB 06126. Statistical analysis was performed using unpaired t tests. (n=5)

(E) DARTS analysis demonstrating PSB 06126-mediated protection of RSAD2 from protease digestion in a concentration-dependent manner.

(F) Body weight changes in both sham and injured mice from vehicle and PSB 06126‑treated mice at day 14 (n = 3).

(G, H, I, J, K) Representative H&E staining of major organs—heart, liver, spleen, lung, and kidney—in both sham and injured mice from vehicle and PSB 06126‑treated mice at day 14 (scale bar, 200 μm).

(L, M, N, O) Complete blood counts including RBC, WBC, PLT, and HGB in both sham and injured mice from vehicle- and PSB 06126‑treated mice at day 14 (n = 3).

(P, Q, R, S) Serum biochemical parameters including alanine aminotransferase (ALT), aspartate aminotransferase (AST), creatinine (CRE), and blood urea nitrogen (BUN), in both sham and injured mice from vehicle- and PSB 06126‑treated mice at day 14 (n=3).

(T) Quantification of inflammatory cells in skin wounds from *Lepr*^db/db^ mice at 1 d post-injury, treated with vehicle or PSB 06126 (n = 5).

(U) ELISA quantification of IL-1β levels in skin wounds from *Lepr*^db/db^ mice at 1 d post-injury, treated with vehicle or PSB 06126 (n = 3).

Within-group comparisons were analyzed using unpaired t tests; **p* < 0.05; NS, not significant.

**Supplementary Tables**

Supplementary Table 1: Demographic details of human victims

| No. | Sex | Age | Injury | Time post injury | Sampling sites | Postmortem interval | Cause of death |
| --- | --- | --- | --- | --- | --- | --- | --- |
| Injury 1 | M | 28 | Stab wound | Instantaneous death | Forearm | 2d | Traumatic shock |
| Injury 2 | M | 65 | Stab wound | 21h | Dorsum | 2d | Hemorrhagic shock |
| Injury 3 | F | 63 | Traffic accident | 3h 50min | Lower leg | 3d | Traumatic shock |
| Injury 4 | F | 54 | Stab wound | 3d | Upper abdomen | 3d | Hemorrhagic shock |
| Injury 5 | M | 37 | Traffic accident | Instantaneous death | Forearm | 3d | Traumatic shock |
| Injury 6 | F | 54 | Traffic accident | 1d | Forearm | 2d | Traumatic shock |

Supplementary Table 2: Primer sequences of RT-qPCR

| Primer | Sequence (5’-3’) |
| --- | --- |
| *Nlrp3*-F | CTCCAACCATTCTCTGACCAG |
| *Nlrp3*-R | ACAGATTGAAGTAAGGCCGG |
| *Il-1β*-F | TGCCACCTTTTGACAGTGATG |
| *Il-1β*-R | TTCTTGTGACCCTGAGCGAC |
| *Il-6-*F | CTTCTTGGGACTGATGCTGGT |
| *IL-6*-R | GACTCCAGCTTATCTCTTGGTTG |
| *Tnf-α-*F | AGGGGATTATGGCTCAGGGT |
| *Tnf-α-*R | AGCTCAGCTCCGTTTTCACA |
| *Cmpk2-*F | CCGGCACAGGAACCTCATC |
| *Cmpk2-*R | TGGAACACCGCAGAGTTAAGC |
| *Rsad2*-F | AGCTACAGGGACATTCACGC |
| *Rsad2*-R | GCTGGGACCATGAACAAACAG |
| *Gapdh*-F | CTTTGTCAAGCTCATTTCCTGG |
| *Gapdh*-R | TCTTGCTCAGTGTCCTTGC |

Supplementary Table 3: Primer sequences of RT-qPCR

| Primer | Sequence (5’-3’) |
| --- | --- |
| *D-loop-*F | AATCTACCATCCTCCGTGAAACC |
| *D-loop*-R | TCAGTTTAGCTACCCCCAAGTTTAA |
| *Tert*-F | CTAGCTCATGTGTCAAGACCCTCTT |
| *Tert*-R | GCCAGCACGTTTCTCTCGTT |
| *B2m*-F | ATGGGAAGCCGAACATACTG |
| *B2m*-R | CAGTCTCAGTGGGGGTGAAT |
| *non-NUMT*-F | CTAGAAACCCCGAAACCAAA |
| *non-NUMT*-R | CCAGCTATCACCAAGCTCGT |

Supplementary Table 4: The plasmid used in the study

| Plasmid Designation | Vector | Label | Application |
| --- | --- | --- | --- |
| HA-YN155-Rsad2 | pCMV | HA | Bimolecular fluorescence complementation |
| HA-YN155-Vector | pCMV | HA |  |
| Flag-YC155-Cmpk2 | pCMV | Flag |  |
| Flag-YC155-Vector | pCMV | Flag |  |
| Rsad2-pet-22b-next | pET | His | Protein purification,  GST-Pull Down |
| Cmpk2-pGEX-6P-1 | pGEX | GST |  |
| His-Vector | pcDNA3.1 | His | Transfection,  Molecular truncation |
| His-CMPK2^1~488^ | pcDNA3.1 | His |  |
| His-CMPK2^75~488^ | pcDNA3.1 | His |  |
| His-CMPK2^257~488^ | pcDNA3.1 | His |  |
| Flag-RSAD2 | pcDNA3.1 | Flag |  |

Supplementary Table 5

| Primer | Sequence (5’-3’) |
| --- | --- |
| *Cox1-*F | GCCCCAGATATAGCATTCCC |
| *Cox1*-R | GTTCATCCTGTTCCTGCTCC |
| *Cytb*-F | ACCCGCCCCATCCAACATCTCAT |
| *Cytb*-R | TTGAGGCTCCGTTTGCGTGT |
| *mt-Atp8*-F | CAAACATTCCCACTGGCACC |
| *mt-Atp8*-R | TTGTTGGGGTAATGAATGAGGC |
| *mt-Nd1-*F | TCCGAGCATCTTATCCACGC |
| *mt-Nd1-*R | GTATGGTGGTACTCCCGCTG |
| *mt-N31-*F | AGTTGCATTCTGACTCCCCC |
| *mt-Nd3-*R | CTTGTAGGGTCGAATCCGCA |
| *mt-Nd5-*F | ACCCAATCAAACGCCTAGCA |
| *mt-Nd5-*R | GGACTGGAATGCTGGTTGGT |

Supplementary Table 6

| Primer | Sequence (5’-3’) |
| --- | --- |
| *ISRE-1-*F | CTGCGCGTCCTAAGG0TTCAG |
| *ISRE-1*-R | TCACAAGGAGGAGGGAAACAC |
| *ISRE-2*-F | CCTGCAAGGATGCAGCTATATG |
| *ISRE-2*-R | ATCTGTCCTGATGACGCTGC |
| *ISRE-3*-F | ATTTCTCTGTTGTGTTTGCCTGG |
| *ISRE-3*-R | ATGTTGACCTTGACAGTGCC |
| *ISRE-4-*F | ACCCGCCCCATCCAACATCTCAT |
| *ISRE-4-*R | TTGAGGCTCCGTTTGCGTGT |
| *ISRE-5-*F | GAGAACACAGAGCTCCCAACG |
| *ISRE-5-*R | GTTCCTTGGGGCAACACATCT |

**Supplementary Materials and Methods**

Spatial Transcriptomics and Bioinformatics Analysis

Spatial transcriptomic profiling was performed by Novogene Co., Ltd. Fresh skin tissues from mice at multiple time points after injury (12 h, 1 d, 3 d, 5 d, 7 d, 10 d, and 14 d) were snap-frozen in liquid nitrogen, embedded in OCT, and subjected to 10× Visium spatial transcriptome sequencing. Bioinformatic analyses were conducted as follows. (Ⅰ) Gene expression matrices were processed using Seurat (v4.3.0.1) in R. After SCT normalization and integration of multiple sections, dimensionality reduction was performed using PCA (30 components) and UMAP. Clustering was carried out via the Louvain algorithm at a resolution of 0.8, yielding 11 distinct clusters. Cell types were annotated based on canonical marker genes and further validated through manual curation. (Ⅱ) Differential gene expression analysis was performed using the FindMarkers function in Seurat (|log_2_^FC^|＞1, *p* < 0.05). Functional enrichment and GSEA were conducted with clusterProfiler (v4.6.2). (Ⅲ) Cell-cell communication networks were inferred using CellChat (v2.1.2) based on the CellTalkDB database, filtering ligand–receptor pairs with *p* < 0.05. (Ⅳ) A protein-protein interaction (PPI) network of the top 20 differentially expressed genes in *Rsad2*^+^ macrophages was constructed using STRING (v12.0; confidence score > 0.4) and visualized in Cytoscape.

Isolation of skin mononuclear cells (SMNCs)

SMNCs were isolated from single-cell suspensions using Percoll (Cytiva, #103432286) density gradient centrifugation. Discontinuous gradients were prepared with upper (1.070 g/mL) and lower (1.085 g/mL) Percoll layers. The cell suspension was layered on top and centrifuged at 1000×g for 25 min at room temperature with low acceleration/deceleration settings. SMNCs were collected from the intermediate layer, washed with DPBS, and processed for downstream applications.

Multicolor Flow Cytometry

SMNCs were resuspended at 5×10⁶ cells per tube in DPBS. Cells were stained with Fixable Viability Stain 700 (BD Horizon, #3305630) for 10 min at room temperature in the dark. After washing with staining buffer containing BSA, cells were incubated with Purified Rat Anti-Mouse CD16/CD32 (BD Horizon, #553141) for 5 min at 4°C to block. Surface staining was performed using a cocktail of the following antibodies for 30 min at 4°C in the dark: BV510 anti-mouse CD3 (BD Horizon, #563024), APC-Cy7 anti-mouse CD45 (BD Horizon, #557659), PE anti-mouse F4/80 (BD Horizon, #565410), PerCP-Cy5.5 anti-mouse CD19 (BD Horizon, #551001), and PE-Cy7 anti-mouse Ly-6C (BD Horizon, #506593). Cells were washed twice and fixed using fixation buffer from the Intracellular Fixation & Permeabilization Kit (Elabscience, #E-CK-A109) for 30 min at 4°C. For intracellular RSAD2 staining, cells were permeabilized with permeabilization working solution and incubated with anti-RSAD2 antibody (Novus, #NBP3-07918) for 30 min at 4°C. After washing, cells were stained with Donkey anti-mouse IgG Alexa Fluor 488 (Thermo, #A-21202) for 30 min at 4°C. Finally, cells were washed and resuspended in staining buffer for acquisition on a BD flow cytometer. Data were analyzed with FlowJo (v10.9, BD Biosciences) and t-SNE dimensionality reduction was performed using the built-in plugin.

Multiplexed immunohistochemistry (mIHC)

Multiplexed IHC was performed on 4-μm-thick formalin-fixed paraffin-embedded tissue sections using a sequential staining approach with the TSA 5-color kit (abs50013, Absinbio). After deparaffinization, slides were incubated with anti-CD11b antibody (#133357, Abcam) for 30 min, followed by incubation with an HRP-conjugated secondary antibody (abs50015-02, Absinbio) for 10 min. Signal was developed with TSA 570 for exactly 10 min according to the manufacturer’s instructions. Following TBST washes, antigen retrieval was performed in preheated citrate buffer using microwave irradiation. Slides were cooled to room temperature in the same buffer and washed with Tris buffer between all steps. The staining cycle was repeated sequentially for the following antibody/TSA pairs: anti-Ly6G (#87048, CST) / TSA 520, anti-F4/80 (#70076, CST) / TSA 700, and anti-RSAD2 (#28089-1, Proteintech) / TSA 620. Finally, sections were counterstained with DAPI, rinsed in distilled water, and coverslipped. Slides were air-dried and scanned using a Pannoramic MIDI II slide scanner. Image analysis was performed with Indica Halo software.

Primary Macrophage Isolation

Bone marrow-derived macrophages (BMDMs) were isolated from the femur and tibia of mice. Bone marrow was flushed with PBS using a syringe, passed through a 70 μm cell strainer, and treated with red blood cell lysis buffer. Cells were resuspended in complete medium supplemented with 20 ng/mL M-CSF and cultured. On day 3, half of the medium was replaced with fresh M-CSF-containing medium. Differentiated adherent macrophages were obtained on day 7, identified by flow cytometry. Peritoneal macrophages (PMs) were elicited by intraperitoneal injection of 3 mL of sterile 4% thioglycolate medium three days prior to extraction. After CO_2_ asphyxiation, the peritoneal cavity was lavaged repeatedly with ice-cold RPMI-1640 containing 10% FBS. Lavage fluid was centrifuged at 400×g for 10 min at 4°C. Cells were washed with PBS, resuspended in complete medium, and plated for 2 h. Non-adherent cells were removed by replacing the medium.

Cell Culture and Treatment

All cell lines were obtained from Procell Life Science & Technology Co., Ltd and tested negative for mycoplasma and chlamydia. Primary macrophages and THP-1 cells were cultured in RPMI-1640 supplemented with 10% FBS and 1% P/S. HEK-293T cells were maintained in DMEM-H with 10% FBS and 1% P/S. HL-60 cells were cultured in specialized medium (#TCH-G204, HyCyte). For differentiation, THP-1 cells were treated with 200 ng/mL PMA (#HY-18739, MCE) for 24 h, then replaced with fresh medium for another 12 h. HL-60 cells were differentiated using 1.25% DMSO (#HY-Y0320, MCE) and 1 μM retinoic acid (#HY-14649, MCE) for 3-5 days. For inflammasome activation, BMDMs were treated with LPS (500 ng/mL, 5h) alone (#S1732, Beyotime), ATP (5 mM) alone (#56-65-5, MCE) , or LPS (500 ng/mL, 4 h) followed by ATP (5 mM, 1 h), as indicated. To establish injury models, macrophages were treated with 250 μM H₂O₂ (#H1009, Sigma), 500 ng/mL LPS, or subjected to mechanical scratch, each for 12 h. Cells were washed with PBS after treatment. To assess CMPK2 protein half-life, BMDMs were treated with 20 μg/mL cycloheximide (#GC17198, GLPBIO) for 0-24 h. To investigate degradation pathways, 10 μM MG132 (#GC10383, GLPBIO) or 10 μM chloroquine (#HY-17589A, MCE) for 6 h was added 1 h before LPS (500 ng/mL, 4 h) and ATP (5 mM, 1 h) stimulation under LPS+ATP conditions. For STING activation, BMDMs were treated with 20 μM cGAMP diammonium (#HY-110385A, MCE) and LPS for 6 h, followed by ATP for 1 h. All cell lines were tested negative for Mycoplasma.

Immunofluorescence Staining

Tissue sections were baked at 65 °C for 15 min, deparaffinized, rehydrated, and subjected to antigen retrieval. Cultured cells were fixed with 4% PFA for 20 min and permeabilized with 0.1% Triton X-100 for 10 min. Both samples were blocked with 5% donkey serum for 2 h at room temperature and then incubated overnight at 4 °C with the following primary antibodies: anti-F4/80 (#2739557, Invitrogen), anti-RSAD2 (#DF12227, Affinity), anti-CD31(#AF3628,

R&D Systems), anti-NLRP3 (#15101S, CST), anti-CMPK2 (#PA5-34461, Invitrogen), or anti–p-IRF3 (#5483, CST). After washing, samples were incubated with fluorophore-conjugated secondary antibodies for 2 h at room temperature. Nuclei were stained with DAPI, and slides were mounted with antifade mounting medium. Images were acquired using a ZEISS slide scanner or confocal microscope and analyzed with Image J.

AAV Injection *In Vivo*

After one week of acclimation, mice were anesthetized and the dorsal hair was removed. Under isoflurane anesthesia, 300 μL of AAV suspension (1×10¹² vg/mL) was injected subcutaneously into the lifted midline dorsal skin. Control mice received AAV9-CD68- Vector-GFP (AAV-Vector), while experimental mice were injected with AAV9-CD68-Rsad2-GFP (AAV-Rsad2^+^). Transfection efficiency was assessed 3~4 weeks post-injection.

Assessment of Inflammatory Cell Infiltration

Inflammatory cell infiltration in skin wound areas was evaluated using HE staining, mast cell staining, and IHC staining on paraffin-embedded sections. Skin tissues were fixed, dehydrated, transparentized, and embedded in paraffin, and 5-μm-thick sections were prepared. After deparaffinization and rehydration. For HE staining, sections were stained with hematoxylin, differentiated in hydrochloric acid alcohol, counterstained with eosin, dehydrated through an ethanol series, transparentized in xylene, and mounted with neutral resin. For mast cell staining, rehydrated sections were stained with toluidine blue working solution for 15 min, differentiated in 95% ethanol until mast cells appeared purplish-blue against a pale background, dehydrated, transparentized, and mounted. For IHC, antigen retrieval was performed using citrate-based acidic buffer or Tris-EDTA (pH 9.0) alkaline buffer via microwave heating. After blocking endogenous peroxidase and washing, sections were incubated overnight at 4 °C with anti–F4/80 (#6640, Abcam ) or anti–MPO (#22225-1-AP, Proteintech). Sections were then incubated with HRP secondary antibodies, developed with DAB, counterstained with hematoxylin, dehydrated, and mounted. Positive inflammatory cells within the wound area were quantified using Image J.

Masson’s Trichrome Staining

Collagen deposition in skin tissues was evaluated using Masson’s Trichrome Stain Kit (#G1340, Solarbio). Briefly, paraffin sections were deparaffinized and rehydrated, followed by nuclear staining with Weigert’s iron hematoxylin for 5 min. After differentiation and blueing in running water, sections were stained in Ponceau-acid fuchsin solution for 5 min, differentiated in phosphomolybdic acid solution, and then counterstained with aniline blue for 20 sec. Finally, sections were dehydrated, cleared, and mounted. Collagen fibers were imaged and quantitatively analyzed for positive area and fiber thickness using ImageJ.

RNA Extraction and Quantitative RT-PCR

Total RNA was isolated from skin tissues using TRIzol reagent according to the manufacturer's instructions. Briefly, tissues were homogenized in TRIzol with steel beads using a high-throughput tissue grinder. After chloroform separation and isopropanol precipitation, the RNA pellet was washed three times with 75% ethanol, air-dried, and dissolved in DEPC-treated water. RNA concentration and purity were determined spectrophotometrically at OD260/280, and samples with ratios between 1.8 and 2.0 were adjusted to 250 ng/μL. Genomic DNA was removed using the PrimeScript RT Reagent Kit with gDNA Eraser (#RR047A, Takara). qPCR was performed using TB Green Premix Ex Taq II (#RR820A, Takara) on a LightCycler 480 system. Gene-specific primers (Supplementary Table 2) were designed using PrimerBank and IDT DNA. Relative mRNA expression levels were calculated using the 2^–ΔΔCt^ method with normalization to a reference gene.

Isolation of Mitochondrial and Cytosolic Fractions

Mitochondrial and cytosolic fractions were isolated from macrophages using the Mitochondrial Isolation Kit (#C3601, Beyotime). Briefly, cells were collected, washed with PBS, and resuspended in ice-cold isolation reagent containing PMSF and phosphatase inhibitors. After incubation on ice for 10-15 min, cells were homogenized using a glass homogenizer. Homogenization efficiency was monitored by trypan blue staining, with a >50% cell breakage rate considered acceptable. The homogenate was centrifuged at 1000×g for 10 min at 4°C, and the supernatant was further centrifuged at 11,000×g for 10 min to pellet the mitochondrial fraction. The resulting supernatant was subjected to an additional centrifugation at 12,000×g for 10 min to obtain the cytosolic fraction. Mitochondria were resuspended in storage buffer for functional assays or lysed for protein analysis.

Assessment of Macrophages Function

Lysosomal function, cytosolic Ca²⁺ and total ROS of BMDMs were evaluated by flow cytometry. (Ⅰ) Lysosomal function was assessed using DQ™ Green BSA (#D12050, Invitrogen). Cells were incubated with 10 μg/mL DQ-BSA at 37 °C for 6 h, washed, and resuspended in staining buffer for flow cytometry analysis in the FITC channel. (Ⅱ) Cytosolic Ca²⁺ levels were measured with the Fluo-4 Calcium Assay Kit (#S1061S, Beyotime). Cells were incubated with 1 mL Fluo-4 working solution at 37 °C for 30 min in the dark, washed, and analyzed via flow cytometry (Ex/Em = 490/525 nm). (Ⅲ) Total ROS levels were detected using the DCFH-DA probe. Cells were loaded with 1 mL DCFH-DA working solution at 37 °C for 30 min, washed with serum-free medium, and fluorescence was measured in the FITC channel. Data were analyzed using FlowJo. (Ⅳ) Phagocytosis was assessed by confocal microscopy using pHrodo™ Red Zymosan A BioParticles (#P35364, Invitrogen). BMDMs were incubated with 100 μg/mL BioParticles for 1 h; The percentage of pHrodo⁺ cells was quantified. (Ⅴ) Bactericidal activity was evaluated by gentamicin protection assay. BMDMs were infected with E. coli (MOI = 10) for 1 h, followed by 100 μg/mL gentamicin for 45 min. After lysis, serial dilutions were plated on LB agar and CFU were counted after overnight incubation.

Assessment of Mitochondrial Function in Macrophages

Mitochondrial function of BMDMs were assessed using the following assays after respective treatments. (Ⅰ) Mitochondrial membrane potential was measured with the JC-1 Assay Kit (#C2006, Beyotime). Cells were incubated with 1 mL JC-1 working solution at 37 °C for 30 min in the dark, washed with assay buffer, and analyzed by flow cytometry using PE and FITC channels. (Ⅱ) Mitochondrial permeability transition pore (mPTP) opening was detected using the mPTP Assay Kit (#C2009S, Beyotime). Cells were stained with 1 mL Calcein AM working solution containing 10 μL CoCl₂ for 30 min at 37 °C, followed by nuclear staining with Hoechst 33342. Samples were observed under a fluorescence microscope. (Ⅲ) Mitochondrial ROS levels were evaluated using MitoSOX™ Red (#M36006, Invitrogen). Cells were incubated with 1 μM MitoSOX working solution at 37 °C for 30 min, washed, and then stained with 200 nM MitoTracker® Red and Hoechst 33342. Imaging was performed within 2 h using a confocal microscope (Ex/Em = 488/510 nm for MitoSOX; 579/599 nm for MitoTracker).

Detection of Newly Synthesized mtDNA (EdU Assay)

BMDMs were cultured overnight and treated with 500 ng/mL LPS and 2 mM EdU (#KTA2030, Abbkine) for 6 h. To examine the colocalization of newly synthesized mtDNA with ASC specks, ATP was added during the last hour of treatment. Cells were then fixed with 4% PFA, permeabilized with 0.1% Triton X-100, and incubated with the Click-iT reaction mixture for 2 h at room temperature in the dark. After blocking with 1% BSA and 5% normal goat serum, samples were immunostained with an anti-ASC antibody overnight at 4 °C, followed by incubation with a fluorescent secondary antibody. Nuclei were counterstained with DAPI before imaging.

Cytokine Measurement by ELISA

Cytokine levels (IL-6, IL-1β, TNF-α, and IFN-β) in skin tissues were measured using commercial ELISA kits (Elabscience; IL-6: #E-HSEL-0003, IL-1β: #E-HSEL-0001, TNF-α: #E-HSEL-0009, IFN-β: #E-EL-M0033). Tissue homogenates were prepared in ice-cold PBS supplemented with protease inhibitors and centrifuged to obtain supernatants. Samples and serially diluted standards were added to pre-coated plates and incubated at 37 °C for 90 min. After washing, biotinylated detection antibody was applied and incubated for 1 h, followed by HRP-conjugated streptavidin for 30 min. The reaction was developed with TMB substrate and terminated with stop solution. Absorbance was read at 450 nm, and cytokine concentrations were interpolated from a four-parameter logistic standard curve. Statistical analyses were performed using GraphPad Prism version 9.

ox-mtDNA Measurement (8-OHdG Method)

The level of oxidized mitochondrial DNA (ox-mtDNA) was determined by quantifying 8-hydroxy-2′-deoxyguanosine (8-OHdG) using a commercial ELISA kit (#D7510009, BBI). Mitochondrial and cytosolic fractions were isolated from BMDMs or SMNCs as described above. Samples and standards were added to the plate, followed by biotinylated detection antibody. After incubation at 37 °C for 90 min and washing, HRP-conjugated streptavidin was added and incubated for 30 min at 37 °C. The reaction was stopped with 50 μL of stop solution, and absorbance was measured at 450 nm. The concentration of 8-OHdG was determined based on a standard curve.

Cell transfection

Plasmids were transfected into cells using Lipofectamine™ 3000 Transfection Reagent. Briefly, cells were seeded at an appropriate density and transfected at 70-80% confluency. For each transfection, plasmid DNA was diluted in Opti-MEM® medium (#31985070, Gibco) and mixed with P3000™ Enhancer Reagent. Separately, Lipofectamine™ 3000 reagent was diluted in Opti-MEM®. The two solutions were combined, incubated for 10-15 min at room temperature to form complexes, and then added to the cells. After gentle mixing, cells were cultured for 24-48 h for further analysis. Plasmids used in this study are listed in Supplementary Table 4.

Co-immunoprecipitation (Co-IP)

Co-IP was performed using the IP/Co-IP Kit (#88828, Thermo Scientific). Briefly, cells were lysed in Co-IP lysis buffer (PR20037, Proteintech) containing PMSF Cocktail (HY-K0010, MCE) and phosphatase inhibitors. After centrifugation, supernatants were collected and protein concentration was determined by BCA assay. For non-conjugated antibodies (e.g., anti-RSAD2), protein lysates were incubated with antibodies and magnetic beads overnight at 4°C with rotation. For pre-conjugated magnetic beads (e.g., Anti-His magnetic beads [#L-1015S, BIOLINK] or Anti-Flag magnetic beads [#L-1012S, BIOLINK]), the beads were directly incubated with protein lysates overnight at 4°C with rotation. Following incubation, all beads were collected through Magnetic Separation Stand (#Z5342, Promega) and washed three times. Then, the beads were resuspended and heated in loading buffer and analyzed by western blotting using antibodies against CMPK2, His-Tag (#PTM-5614, PTM Bio), Flag-Tag (#PTM-8075, PTM Bio), Ubiquitin (#PTM-1107, PTM Bio), Acetyllysine (#PTM-105RM, PTM Bio), Ubiquitin-K48 (#PTM-7205, PTM Bio), Ubiquitin-K63 (#PTM-7228, PTM Bio), Phospho-Tyrosine Rabbit mAb (#PTM-702RM, PTM Bio), or Phospho-Threonine (#PTM-705RM, PTM Bio). VeriBlot for IP Detection Reagent (#1035165-1, Thermo) was used for secondary detection. Data were analyzed by Image J.

Molecular Docking Analysis

Protein sequences were obtained from the UniProt database (https://www.uniprot.org/). The three-dimensional structures of CMPK2 and Csnk2a2 were predicted using AlphaFold2, while the crystal structure of RSAD2 (PDB: 6Q2P) was employed due to its high resolution and wild-type properties. All structures were preprocessed through hydrogen addition, hydrogen bond optimization, and energy minimization. Protein–protein docking was performed using the Schrödinger software suite with the Protein–Protein Docking module. CMPK2 was set as the receptor, and RSAD2 or Csnk2a2 as the ligand. Thirty conformational poses were generated and subjected to clustering analysis. The optimal pose was selected for interaction site evaluation. The binding free energy was calculated using the MM-GBSA method to assess binding affinity.

Identification of RSAD2–CMPK2 Interaction Domains

To identify the key domains mediating the interaction between CMPK2 and RSAD2, we first analyzed the domain architecture of CMPK2 using NCBI (https://www.ncbi.nlm.nih.gov/), SMART (https://smart.embl.de/), and UniProt databases. Based on the consensus predictions, we constructed a series of CMPK2 truncation mutants: full-length His‑CMPK2^1-488^ and two truncated variants, His‑CMPK2^75-488^and His‑CMPK2^257-488^, with an empty His-tag vector serving as a negative control. These constructs were co-transfected with Flag‑RSAD2 into BMDMs. Subsequently, Co-IP was performed using anti-Flag magnetic beads. The immunoprecipitated complexes were eluted and analyzed by western blotting with anti-His and anti-Flag antibodies to determine the interaction domains.

CMPK2 Kinase Activity Assay

CMPK2 kinase activity was assessed through two complementary methods using immunopurified His-tagged CMPK2 protein from BMDMs. First, an *in vitro* kinase assay measured ATP consumption: purified CMPK2 was incubated with dCMP and ATP in optimized buffer (50 mM Tris-HCl, 10 mM MgCl_2_, 2 mM DTT, 50 mM NaCl, 10 μM ATP, and 5 mM dCMP), and residual ATP was quantified using a luminescent kinase assay kit (#S0150S, Beyotime). Second, substrate conversion was directly evaluated by ELISA: parallel reaction products were analyzed to determine levels of dCMP and dCDP, with activity expressed as the dCDP/(dCMP + dCDP) ratio.

Mass Spectrometric Analysis of CMPK2-Interacting Proteins

To identify proteins that interact with CMPK2, GST pull-down assays combined with LC-MS/MS were performed. Purified GST-CMPK2 fusion protein was incubated with protein lysates from LPS+ATP-stimulated primary macrophages, using GST-tagged protein as a control. The captured protein complexes were separated by SDS-PAGE and subjected to in-gel tryptic digestion. The resulting peptides were analyzed using a Thermo UltiMate 3000 RSLCnano system with a C18 column in data-dependent acquisition mode (MS1 resolution: 60,000; MS2 resolution: 15,000; collision energy: 28%). Raw data were processed using MaxQuant (v1.6.6.0) against the UniProt mouse protein database, with an FDR threshold of 1%. Proteins identified in the GST control group were excluded, and specific binding candidates were selected for further bioinformatic analysis using GO, KEGG, Pfam, and subcellular localization databases. Potential interactors were subsequently validated by Co-IP.

Virtual Screening and *In Vivo* Validation Targeting RSAD2

Based on the crystal structure of mouse RSAD2 (PDB ID: 6Q2P), a grid file centered on the CTP site (coordinates: 4.553, 16.966, 52.042 Å; box dimensions: 20.316 Å³) was generated. Virtual screening of the NeoraBio Bioactive Compound Library (35.1K compounds) against this site was performed using Schrödinger Maestro 11.4, and the binding poses of the top‑10 scoring compounds were visualized with PyMOL. Candidate compounds, including PSB‑06126 (#B7113, APExBIO), PDK1 inhibitor (#A3706, APExBIO), and LCA (#HY‑N0457A, MCE), were evaluated in *WT* and *Lepr*^db/db^ mouse skin wound models. Mice received daily intraperitoneal injections of each compound (10 mg/kg) or vehicle, starting 3 days before modeling and continuing until tissue collection.

Drug Affinity Responsive Target Stability assay (DARTS)
The DARTS assay was performed using BMDMs from *WT* mice. LPS‑treated BMDMs were lysed with M‑PER reagent (#78501, Thermo). Lysates were centrifuged at 12,000×g for 15 min at 4 °C. The supernatants were collected, and protein concentration was determined by BCA assay and adjusted to 2 µg/µL with 1× TNC buffer (#T4301, Solarbio). Equal amounts of protein lysate were incubated with 100 µM PSB 06126 or an equivalent volume of vehicle control at 4 °C with rotation for 90 min. Each sample was then divided into aliquots and digested with pronase (#P5147, Merck) at different enzyme‑to‑protein ratios (0, 1:100, 1:500, 1:1000, 1:2000) for 20 min at room temperature. Reactions were terminated by adding loading buffer and boiling at 100 °C for 5 min. Protein stability was subsequently analyzed by SDS‑PAGE and Western blotting.

Immunoprecipitation of Inflammasome Complexes

BMDMs were lysed in Co-IP buffer containing protease and phosphatase inhibitors. Lysates were adjusted to a total protein mass of 5 mg in 300 μL and incubated with anti-ASC (#67824, CST) or anti-cGAS (#31659S, CST) antibodies for 2 h at 4 °C with rotation, followed by the addition of pre-washed agarose beads and overnight incubation. Immune complexes were eluted under non-denaturing conditions using elution buffer and immediately neutralized. Eluates were supplemented with DTT. DNA was extracted from the eluates using proteinase K and buffer system-based purification. Precipitated DNA was amplified by qPCR with primers targeting mitochondrial genes (e.g., D-loop, Cox1); Tert was used for normalization. Precipitated mtDNA levels were quantified as described in Supplementary Table 5.

Chromatin Immunoprecipitation (ChIP) Assay

To examine the binding of transcription factor IRF-3 to the *Rsad2* promoter in macrophages, potential IRF-3 binding sites within interferon-stimulated response elements were predicted using the JASPAR database (http://jaspar.genereg.net/) (Supplementary Table 6). ChIP was performed using the SimpleChIP® Plus Sonication Chromatin IP Kit (#56383, CST). BMDMs were stimulated with LPS and ATP, cross-linked with formaldehyde, and quenched with glycine. Cells were lysed and chromatin was fragmented by sonication. Chromatin fragments were immunoprecipitated overnight at 4 °C with antibodies against IRF-3 (#11312-1-AP, Proteintech), Histone H3 (#14269, CST), or IgG. Complexes were captured with Protein A/G magnetic beads, washed with low-salt, high-salt, LiCl, and TE buffers, and eluted. Cross-links were reversed by heating at 65 °C for 4 h, followed by proteinase K digestion. DNA was purified using a commercial kit (#D0033, Beyotime) and enriched regions were analyzed by agarose gel electrophoresis.

Protein Extraction and Western Blot Analysis

Protein lysates were extracted from tissue samples or cultured cells. Tissue samples were homogenized in RIPA buffer containing protease and phosphatase inhibitors using a tissue homogenizer, while cell samples were lysed directly in RIPA buffer followed by ultrasonic disruption. Lysates were centrifuged and supernatants were collected. Protein concentration was determined by BCA assay, and equal amounts of protein were separated by SDS-PAGE, transferred to PVDF membranes, and blocked with 5% non-fat milk. Membranes were incubated with primary antibodies overnight at 4°C, followed by HRP-conjugated secondary antibodies, and signals were detected using ECL. Band intensities were quantified with Image J. Antibodies included anti-RSAD2, CMPK2, NLRP3, GAPDH, NF-κB (#10745, Proteintech), phospho-NF-κB (#3033, CST), NF-κB (#10745, Proteintech), phospho-NF-κB (#3033, CST), TGF-β (#3711S, CST), COL1A1 (#72026S, CST), Fibronectin (#ab2413, Abcam), p-Smad2/3 (#251795, Zenbio), Smad2/3 (#382472, Zenbio), α-SMA (#ab5694, Abcam), Vimentin (#10366-1-AP, Proteintech), anti-cGAS (#31659S, CST), IRF-3 (#11312-1-AP, Proteintech), TBK1 (#67211-1-lg, Proteintech), STING (#19851-1-AP, Proteintech), phospho-IRF-3 (#5483, CST), phospho- TBK1 (#29047, CST), phospho- STING (#72971T, CST), Caspase-1 (#AG-20B-0042-C100, AdipoGen), Cleaved-Caspase-1 (#89332T, CST), IL-1β (#GB11113, Servicebio), IL-18 (#10663-1-AP, Proteintech).

Quantification and Statistical Analysis
Protein band intensities from Western blotting were quantified using ImageJ software, normalized to GAPDH, and expressed as relative values with the control group set to 1. For morphological analyses, the number of positive cells and mean fluorescence intensity in skin wound areas were quantified using ImageJ from three randomly selected high-power fields (20× or 40×) per mouse, and the average value was calculated for each sample. Statistical analyses were performed with Prism 9.0 (GraphPad Software). Normality was assessed using the Shapiro‑Wilk test, and homogeneity of variance was evaluated with the F‑test (for t‑tests) or Brown‑Forsythe and Bartlett’s tests (for ANOVA). Comparisons between two groups were made by unpaired Student’s t‑test. Comparisons among three or more groups were analyzed by one‑way ANOVA followed by Tukey’s HSD post‑hoc test for pairwise comparisons. **p*<0.05 was considered statistically significant.
